# Genetic Variants of *CLEC4E* and *BIRC3* in Damage-Associated Molecular Patterns-Related Pathway Genes Predict Non-Small Cell Lung Cancer Survival

**DOI:** 10.3389/fonc.2021.717109

**Published:** 2021-10-06

**Authors:** Lihua Liu, Hongliang Liu, Sheng Luo, Edward F. Patz, Carolyn Glass, Li Su, Lijuan Lin, David C. Christiani, Qingyi Wei

**Affiliations:** ^1^ Department of Pulmonary and Critical Care Medicine, The First Affiliated Hospital of Guangxi Medical University, Nanning, China; ^2^ Duke Cancer Institute, Duke University Medical Center, Durham, NC, United States; ^3^ Department of Population Health Sciences, Duke University School of Medicine, Durham, NC, United States; ^4^ Department of Biostatistics and Bioinformatics, Duke University School of Medicine, Durham, NC, United States; ^5^ Department of Radiology, Duke University Medical Center, Durham, NC, United States; ^6^ Department of Pharmacology and Cancer Biology, Duke University Medical Center, Durham, NC, United States; ^7^ Department of Pathology, Duke University School of Medicine, Durham, NC, United States; ^8^ Departments of Environmental Health and Department of Epidemiology, Harvard School of Public Health, Boston, MA, United States; ^9^ Department of Medicine, Massachusetts General Hospital, Boston, MA, United States; ^10^ Department of Medicine, Duke University Medical Center, Durham, NC, United States

**Keywords:** non-small cell lung cancer, single-nucleotide polymorphism, variant, damage-associated molecular pattern-related pathway, survival

## Abstract

Accumulating evidence supports a role of various damage-associated molecular patterns (DAMPs) in progression of lung cancer, but roles of genetic variants of the DAMPs-related pathway genes in lung cancer survival remain unknown. We investigated associations of 18,588 single-nucleotide polymorphisms (SNPs) in 195 DAMPs-related pathway genes with non-small cell lung cancer (NSCLC) survival in a subset of genotyping data for 1,185 patients from the Prostate, Lung, Colorectal and Ovarian (PLCO) Cancer Screening Trial and validated the findings in another independent subset of genotyping data for 984 patients from Harvard Lung Cancer Susceptibility Study. We performed multivariate Cox proportional hazards regression analysis, followed by expression quantitative trait loci (eQTL) analysis, Kaplan-Meier survival analysis and bioinformatics functional prediction. We identified that two SNPs (i.e., *CLEC4E* rs10841847 G>A and *BIRC3* rs11225211 G>A) were independently associated with NSCLC overall survival, with adjusted allelic hazards ratios of 0.89 (95% confidence interval=0.82-0.95 and *P*=0.001) and 0.82 (0.73-0.91 and *P*=0.0003), respectively; so were their combined predictive alleles from discovery and replication datasets (*P*_trend_=0.0002 for overall survival). We also found that the *CLEC4E* rs10841847 A allele was associated with elevated mRNA expression levels in normal lymphoblastoid cells and whole blood cells, while the *BIRC3* rs11225211 A allele was associated with increased mRNA expression levels in normal lung tissues. Collectively, these findings indicated that genetic variants of *CLEC4E* and *BIRC3* in the DAMPs-related pathway genes were associated with NSCLC survival, likely by regulating the mRNA expression of the corresponding genes.

## Introduction

Lung cancer remains one of the leading causes of cancer-related mortality in the United States. In 2021, it is estimated that there will be more than 235,000 new cases of and nearly 131,000 will die from lung cancer in the United States (1). Histologically, about 85% of lung cancer patients are classified as non-small cell lung cancer (NSCLC), and the majority of these cases present with local progression or distal metastasis at the time of diagnosis (2). Although there are some clinical predictors and newer treatment options for NSCLC, the clinical outcomes remain poor, largely because of the remarkable heterogeneity in the phenotypes of the early NSCLC, showing diverse innate aggressiveness. Some cases with an early stage of the disease have a favorable prognosis and could be spared the unnecessary therapy, while for other patients with an advantaged stage, the five-year survival rate remains poor, despite the use of all the available targeted and immune therapies (3). Therefore, identification of additional prognostic factors for NSCLC-specific survival could add more value to precision medicine of NSCLC.

Damage-associated molecular patterns (DAMPs) are special molecules that are released from the damaged tissues or by the activated immune cells, alerting the organism about the incoming endogenous danger including cancer, inflammation, and tissue repair (4). There is some convincing evidence that DAMPs could recruit specific molecules of the innate and adaptive immune system to tumor microenvironment, ultimately inducing a tumor-targeting immune response (5, 6). Accumulating evidence has revealed the role of various DAMPs in NSCLC progression. For example, one study reported that the high mobility group box 1, one of DAMPs, was found to enhance NSCLC cell migration, leading to metastasis of NSCLC (7). Other studies showed that S100 family members, which also serve as DAMPs, drove NSCLC cell proliferation and invasion (8, 9). Recently, targeting the heat-shock protein family members showed anticancer therapeutic potential for NSCLC patients (10–12). Furthermore, increasing preclinical evidence has indicated that monitoring DAMPs in NSCLC patients could have potential prognostic value and some positive effects on the treatment outcomes (11, 13–15).

As we all know, innate immune interactions in the cancer context include recognition by innate cell populations, dendritic cells and macrophages in response to DAMPs (16). Dying tumor cells would express or release DAMPs for activation of immune cells. That suggests that DAMPs could be the trigger activating tumor innate immunity. But the role of DAMPs in tumor immunity is not completely understood and presents complicated activities of tumor immunity. For example, HMGB1 could trigger both antitumor immunity inflammation and immunotolerance (17).

However, the detection of single DAMP in NSCLC patients may be possible but not be accurate enough for predicting NSCLC prognosis or may cause conflicting results (18). Monitoring of DAMP-associated processes would be more accurate to predict prognosis of NSCLC patients but much difficult (19). Recent studies suggest that identification of single-nucleotide polymorphisms (SNPs) in certain pathway-related genes may help identify novel biomarkers for NSCLC progression and survival (20, 21). Such a post-genome-wide association study (GWAS) strategy with a pathway-based analysis is hypothesis driven, which uses available genotyping data from previously published GWAS datasets to identify functional genetic variants in the targeted biological pathway genes and may clarify possible molecular mechanisms underlying the observed associations with NSCLC survival (22, 23). Therefore, to better understand the value of DAMPs-associated processes in the prognosis of NSCLC survival, we hypothesize that genetic variants of DAMPs-related pathway genes are associated with NSCLC survival, and we tested this hypothesis using genotyping data from two publicly available NSCLC GWASs.

## Materials and Methods

### Study Populations

The discovery genotyping data were derived from a GWAS dataset from the Prostate, Lung, Colorectal and Ovarian (PLCO) Cancer Screening Trial. The age of all the participants were between 55-74 years, who were recruited between 1993 and 2001 in the United States. These participants were randomly assigned to a screening group that received a trial intervention or a control group that received usual care and followed up for 13 years from the time of random assignment (24, 25). During the follow-up 1,185 patients in the screening group were diagnosed with NSCLC, and their detailed clinical information including histologic diagnosis, tumor stage, treatment method, and survival time was recorded. Genomic DNA was extracted from NSCLC patients for genotyping using the Illumina HumanHap240Sv1.0 and HumanHap550v3.0 (dbGaP accession: phs000093.v2.p2 and phs000336.v1.p1) (26–29). All the subjects provided a written informed consent under a protocol approved by the review board of each participating institutions. In contrast, the validation dataset comprised demographic data and clinical information on 984 Caucasian patients with histology-confirmed NSCLC from the Harvard Lung Cancer Susceptibility (HLCS) Study launched in 1992 (30). Genomic DNA from the HLCS NSCLC patients was genotyped using the Illumina Humanhap610‐Quad array. The present study was approved by the Internal Review Board of Duke University School of Medicine (#Pro00054575) and was authorized to have the access so the GWAS datasets by the National Institutes of Health Data Access Committee (dbGaP, #6404). **Supplementary Table S1** showed the detail of clinical characteristics between the PLCO trial and the HLCS study. Since the PLCO discovery data comprised more available covariates than the HLCS validation dataset, we only used the PLCO dataset for further multivariate analyses in the present study.

### Gene Selection and SNP Genotyping

**Figure 1:**
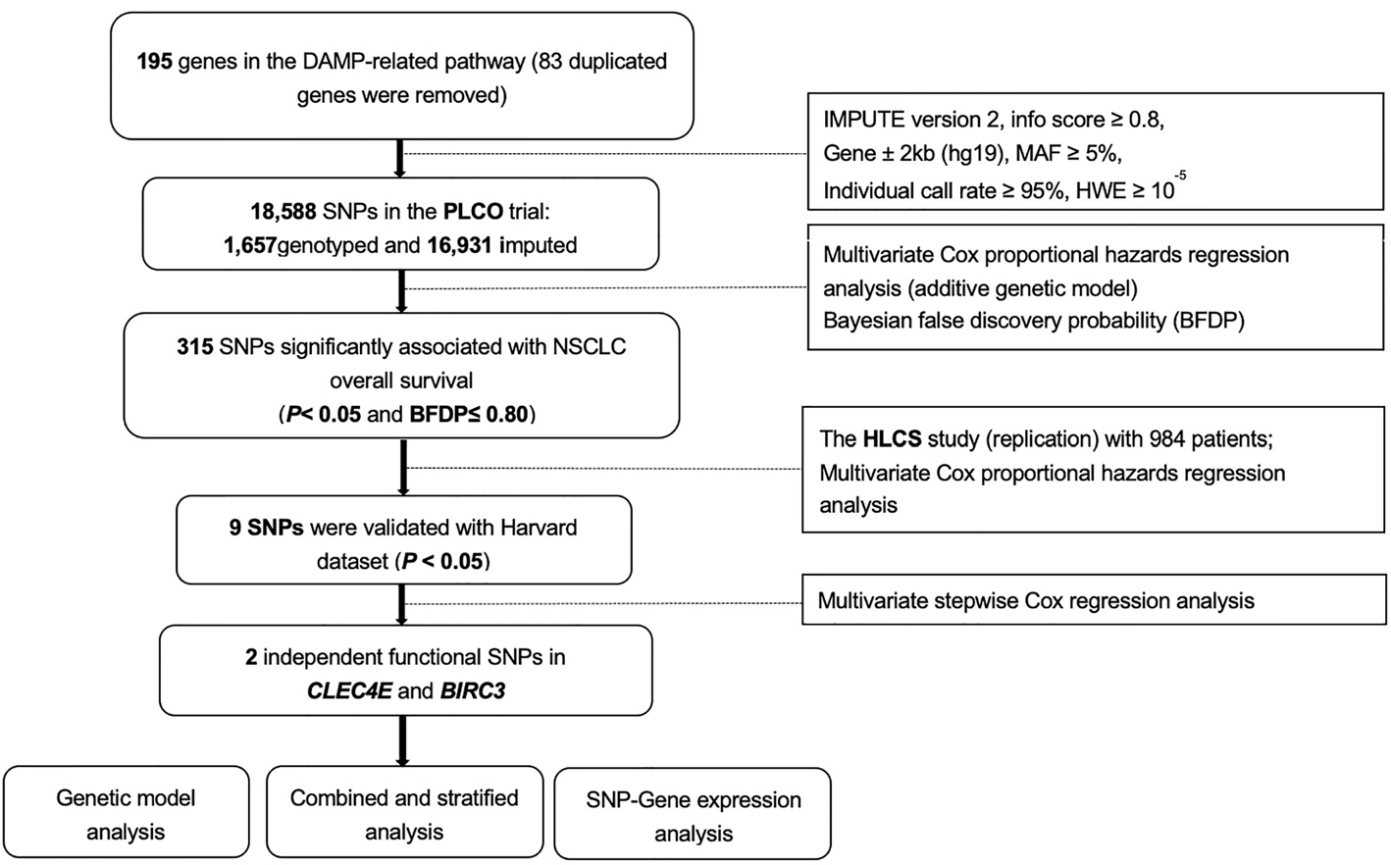
The overall procedures of the present study. DAMP, damage-associated molecular pattern; SNPs, single-nucleotide polymorphisms; MAF, minor allelic frequency; HWE, Hardy-Weinberg Equilibrium; PLCO, The Prostate, Lung, Colorectal and Ovarian Cancer Screening Trial; HLCS, the Harvard Lung Cancer Susceptibility Study; NSCLC, non-small cell lung cancer.

We searched the Molecular Signatures Database of the Gene Set Enrichment Analysis (GSEA) website (http://software.broadinstitute.org/gsea/msigdb/search.jsp) for the genes of a defined pathway, and we identified 195 damage-associated molecular pattern-related pathway genes located only on the autosomes (**Supplementary Table S2**).We extracted SNPs within ± 2 kilobase flanking regions of 195 damage-associated molecular pattern-related pathway genes from the PLCO trial and performed imputation with IMPUTE2 using the reference from the 1,000 Genomes Project European data (phase 3). As a result, a total of 18,588 SNPs (1,657 genotyped and 16,931 imputed) remained for further analysis after quality control [presented in **Figure 1**: located within gene region ± 2kb (hg19), MAF ≥ 5%, HWE P ≥ 10^-5^, individual call rate ≥ 95% (for genotyping SNPs), and imputation info score ≥ 0.8 (for imputed SNPs)]. The distribution of imputation score was presented in **Supplementary Figure S1**.

### Statistical Methods

In the discovery PLCO dataset, we first assessed associations between all available candidate SNPs and NSCLC survival in a single-locus Cox proportional hazards regression analysis using the GenABEL package of R software. SNPs were coded under an additive genetic model according to the number of minor alleles. We performed Cox analyses with adjustment for available covariates in the PLCO trial (including age, sex, histology, smoking status, tumor stage, chemotherapy, surgery, radiotherapy and the first four principal components). The dependent variables included survival time and survival status recorded during the follow-up or at the endpoint of the PLCO trial. In consideration of the high level of linkage disequilibrium (LD) among these candidate SNPs, instead of using the stringent false discovery rate (FDR) method for multiple test correction, we also employed Bayesian false discovery probability (BFDP) with a cutoff value of 0.80 for multiple testing correction to lower the probability of potentially false positive results, as is recommended (31, 32). We assigned a prior probability of 0.10 and a detectable upper boundary HR of 3.0 for an association with variant genotypes or minor alleles of the SNPs with *P*< 0.05 (33).

By using the HLCS validation dataset, we replicated the associations of the discovered significant SNPs associated with NSCLC survival in multivariate Cox regression models comparably with a significance level of *P*< 0.05. To identify independent SNPs associated with NSCLC survival, we subsequently performed a multivariate stepwise Cox regression model with the PLCO trial. In addition to available clinical variables, other 28 previously published SNPs associated with NSCLC survival in the same PLCO dataset were also included in the model for further adjustment.

Next, we performed a meta-analysis to combine the identified SNPs from the PLCO trial with those in the HLCS dataset using the Cochran’s Q statistics and *I* ^2^ to assess the heterogeneity. Since no heterogeneity was observed between discovery trial and validation datasets (*P*_het_> 0.10 and *I* ^2^ < 50%), we implemented fixed-effects model for the meta-analysis. After that, we evaluated cumulative effects of all identified SNPs on NSCLC survival probability. For those remained in the final model, we combined the protective alleles into one variable as a genetic score. NSCLC patients were categorized into four groups (i.e., 0, 1, 2, and 3-4) according to the number of their protective alleles (NPA). For the stratified analyses by subgroups, we calculated inter-study heterogeneity and evaluated possible effect modification or interaction. We constructed a survival prediction model by using the receiver operating characteristic (ROC) curve with the “survival” and “time ROC” packages of R software (version 3.6.2). Sensitivity, specificity, and time-dependent area under the curve (AUC) were used to measure the ability of survival models to predict NSCLC survival in association with both clinical and genetic variables (34). To evaluate the genotype-phenotype correlation between genotypes of identified SNPs and mRNA expression levels of the corresponding genes, we employed expression quantitative trait loci (eQTL) analyses with a general linear regression model using data from the 373 European descendants included in the 1,000 Genomes Project (Phase 3) (35), the genotype-tissue expression (GTEx) project (http://www.gtexportal.org/home; version V8) (36), and The Cancer Genome Atlas (TCGA) database. Additional bioinformatics functional prediction for the tagging SNPs were performed with SNPinfo (37), RegulomeDB (v2.0.3) (38) (http://www.regulomedb.org) and HaploReg V4.1 (39) (http://archive.broadinstitute.org/mammals/haploreg/haploreg.php).

Finally, we depicted the associations between the mRNA expression levels of genes where SNPs are located and NSCLC survival using the Kaplan-Meier (KM) survival curves from the online TCGA database (http://ualcan.path.uab.edu; last updated by 09/23/2019) and from Human Protein Atlas database (https://www.proteinatlas.org/; Version: 20.1). All statistical analyses were performed with SAS software (version 9.4; SAS Institute, Cary, NC), unless specified otherwise.

## Results

### Associations Between SNPs in DAMPs-Related Pathway Genes and NSCLC in the PLCO Trial and the HLCS Study

Baseline characteristics of NSCLC patients from the PLCO trial and the HLCS study are described elsewhere (40), and an overall flowchart of the present study is presented in **Figure 1**. Among the acquired 18,588 SNPs in195 DAMPs-related pathway genes, we found 315 SNPs to be significantly associated with the NSCLC overall survival (OS) in the PLCO trial in an additive model (**Supplementary Table S3A**), of which 9 SNPs remained significant as replicated in the HLCS dataset with multiple test correction by the BFDP (**Supplementary Table S3B**).

### Identification of Independent SNPs Among the Nine Significant SNPs

In stepwise multivariable Cox regression analyses, we assessed effects of the nine validated SNPs on NSCLC survival in the PLCO trial. We then expanded this prediction model with adjustment for 28 previously reported SNPs in the PLCO trial. Finally, we identified two SNPs (*CLEC4E* rs10841847 G>A and *BIRC3* rs11225211G>A) that remained significantly associated with NSCLC OS (*P*=0.019 and 0.012) (**Table 1**). The results of meta-analysis for these two independent SNPs in each and combined datasets are presented in **Table 2**, showing the absence of heterogeneity across these two datasets.

**Table 1:** Two independent SNPs in a multivariate Cox proportional hazards regression analysis with adjustment for other covariables and additional 28 previously published SNPs for NSCLC in the PLCO Trial.

Variables	Category	Frequency	HR (95% CI)^a^	*P* ^a^	HR (95% CI)^b^	*P* ^b^
Age	Continuous	1185	1.04 (1.02-1.05)	<0.0001	1.04 (1.03-1.06)	<0.0001
Sex	Male	698	1.00		1.00	
	Female	487	0.77 (0.66-0.89)	0.0005	0.71 (0.61-0.84)	<0.0001
Smoking status	Never	115	1.00		1.00	
	Current	423	1.70 (1.26-2.26)	0.0004	1.88 (1.39-2.54)	<0.0001
	Former	647	1.74 (1.32-2.29)	<0.0001	1.90 (1.43-2.54)	<0.0001
Histology	Adenocarcinoma	577	1.00		1.00	
	Squamous cell	285	1.20 (1.00-1.45)	0.057	1.21 (1.00-1.48)	0.053
	others	323	1.31 (1.10-1.55)	0.002	1.38 (1.15-1.65)	0.0005
Tumor stage	I-IIIA	655	1.00		1.00	
	IIIB-IV	528	3.00 (2.46-3.65)	<0.0001	3.45 (2.82-4.23)	<0.0001
Chemotherapy	No	639	1.00		1.00	
	Yes	538	0.57 (0.48-0.68)	<0.0001	0.56 (0.47-0.68)	<0.0001
Radiotherapy	No	762	1.00		1.00	
	Yes	415	0.95 (0.81-1.12)	0.569	0.99 (0.83-1.72)	0.880
Surgery	No	637	1.00		1.00	
	Yes	540	0.21 (0.17-0.28)	<0.0001	0.20 (0.15-0.26)	<0.0001
*CLEC4E* rs10841847 G>A	GG/GA/AA	495/544/146	0.88 (0.80- 0.98)	0.019	0.88 (0.79- 0.98)	0.019
*BIRC3* rs11225211 G>A	GG/GA/AA	368/579/248	0.81 (0.71- 0.93)	0.003	0.83 (0.72- 0.96)	0.019

SNP, single-nucleotide polymorphisms; NSCLC, non-small cell lung cancer; PLCO, the Prostate, Lung, Colorectal and Ovarian cancer screening trial; HLCS, Harvard Lung Cancer Susceptibility Study; HR, hazard ratio; CI, confidence interval.

a Adjusted for age, sex, tumor stage, histology, smoking status, chemotherapy, radiotherapy, surgery, and PC1, PC2, PC3, PC4.

b Other additional 28 published SNPs were included for further adjustment: rs779901, rs3806116, rs199731120, rs10794069, rs1732793, rs225390, rs3788142, rs73049469, rs35970494, rs225388, rs7553295, rs1279590, rs73534533, rs677844, rs4978754, rs1555195, rs11660748, rs73440898, rs13040574, rs469783, rs36071574, rs7242481, rs1049493, rs1801701, rs35859010, rs1833970, rs254315, rs425904.rs13040574, rs469783, rs36071574, rs7242481, rs1049493, rs1801701, rs35859010, rs1833970, rs254315, rs425904.

**Table 2:** Associations of two significant SNPs with overall survival of patients with NSCLC in both discovery and validation datasets from two previously published GWASs.

SNPs	Allele^a^	Gene	PLCO (n = 1185)					HLCS (n = 984)			Combined-analysis			
			FDR	BFDP	EAF	HR (95% CI)^b^	*P* ^b^	EAF	HR (95% CI)^c^	*P* ^c^	*P*_het_ ^d^	*I* ^2^	HR (95% CI)^e^	*P* ^e^
rs10841847^f^	G/A	*CLEC4E*	0.61	0.79	0.44	0.88 (0.79-0.97)	0.012	0.42	0.89 (0.80-0.99)	0.039	0.870	0	0.89 (0.82-0.95)	1.41x10^-3^
rs11225211^f^	G/A	*BIRC3*	0.61	0.69	0.17	0.82 (0.72-0.95)	0.006	0.16	0.81 (0.68-0.98)	0.029	0.940	0	0.82 (0.73-0.91)	3.75x10^-4^

SNPs, single-nucleotide polymorphisms; NSCLC, non-small cell lung cancer; GWAS, genome-wide association study; PLCO, the Prostate, Lung, Colorectal and Ovarian cancer screening trial; HLCS, Harvard Lung Cancer Susceptibility Study; EAF, effect allele frequency; HR, hazard ratio; CI, confidence interval; FDR, false discovery rate; BFDP, Bayesian false discovery probability; LD, linkage disequilibrium.

a Reference/effect allele.

b Adjusted for age, sex, stage, histology, smoking status, chemotherapy, radiotherapy, surgery, PC1, PC2, PC3 and PC4.

c Adjusted for age, sex, stage, histology, smoking status, chemotherapy, radiotherapy, surgery, PC1, PC2 and PC3.

d P_het_: P value for heterogeneity by Cochrane’s Q test.

e Meta-analysis in the fix-effects model.

f Imputed SNP in the PLCO trial.

Furthermore, as shown in **Table 3**, we noticed that the *CLEC4E* rs10841847 A allele and the *BIRC3* rs11225211 A allele were protective alleles for NSCLC OS in the PLCO dataset (*P*_trend_= 0.012 and 0.006, respectively) with similar results for NSCLC disease-specific survival (DSS) in the PLCO dataset (*P*_trend_= 0.049 and 0.017, respectively). All identified SNPs in the present study are depicted in Manhattan plots for both PLCO and HLCS (**Supplementary Figure S2** **)** datasets, and regional association plots for these two independent SNPs (also including all SNPs located in their 100-kb flanking regions) are displayed in **Supplementary Figure S3**.

**Table 3:** Associations of the protective alleles of two independent SNPs with OS and DSS of NSCLC in the PLCO Trial.

Alleles		Frequency		OS^a^			DSS^a^		
				Death (%)	HR (95% CI)	*P*	Death (%)	HR (95% CI)	*P*
*CLEC4E* rs10841847 G>A^b^									
GG	361		248 (68.70)		1.00		361 (60.66)	1.00	
GA	597		396 (66.33)		0.88 (0.75-1.04)	0.134	597 (60.30)	0.92 (0.77-1.09)	0.323
AA	217		145 (66.82)		0.77 (0.62-0.95)	0.014	217 (59.91)	0.80 (0.64-0.99)	0.047
Trend test						0.012			0.049
*BIRC3* rs11225211 G>A^c^									
GG	812		559 (68.84)		1.00		812 (61.82)	1.00	
GA	325		206 (63.38)		0.79 (0.67-0.93)	0.004	325 (56.92)	0.80 (0.67-0.95)	0.010
AA	37		23 (62.16)		0.82 (0.54-1.25)	0.351	37 (56.76)	0.86 (0.55-1.33)	0.488
Trend test						0.006			0.017
NPA^d,e^									
0	246		176 (71.54)		1.00		246 (62.20)	1.00	
1	519		342 (65.90)		0.83 (0.69-0.99)	0.042	519 (60.69)	0.87 (0.71-1.05)	0.153
2	323		219 (67.80)		0.74 (0.60-0.91)	0.004	323 (59.75)	0.76 (0.61-0.74)	0.012
3-4	86		51 (59.30)		0.58 (0.42-0.80)	0.0008	86 (54.65)	0.64 (0.46-0.90)	0.009
Trend test						0.0002			0.002
Dichotomized NPA									
0-1	765		518 (67.71)		1.00		765 (61.18)	1.00	
2-4	409		270 (66.01)		0.80 (0.69-0.93)	0.004	409 (58.68)	0.81 (0.69-0.95)	0.009

SNP, single nucleotide polymorphism; NSCLC, non-small cell lung cancer; PLCO, Prostate, Lung, Colorectal and Ovarian cancer screening trial; HR, hazard ratio; CI, confidence interval; OS, overall survival; DSS, disease-specific survival. NPA, number of protective alleles.

a Adjusted for age, sex, smoking status, histology, tumor stage, chemotherapy, surgery, radiotherapy and principal components.

b 10 missing date were excluded.

c 11 missing date were excluded.

d 11 missing date were excluded.

e Protective alleles were CLEC4E rs10841847_A and BIRC3 rs11225211_A.

### Combined Protective Alleles of the Two Independent NSCLC Survival-Associated SNPs

**Figure 2:**
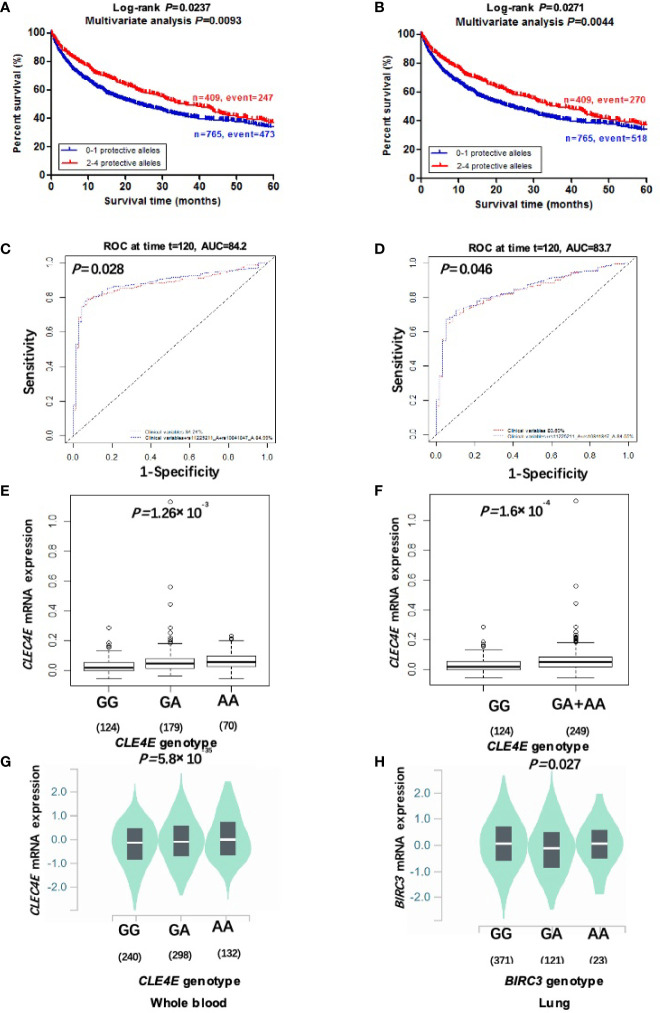
Two independent SNPs in DAMP-related pathway genes predict NSCLC patient’s survival and eQTL analysis. Kaplan-Meier survival curves of the combined risk alleles (0-1 *vs*. 2-4) of *CLEC4E* rs10841847 G>A and *BIRC3* rs11225211 G>Ain the PLCO trial for DSS **(A)** and OS **(B)**. The ten-year NSCLS DSS prediction by ROC curve based on clinical variables plus protective alleles **(C)**. The ten-year NSCLS OS prediction by ROC curve based on clinical variables plus protective alleles **(D)**. The correlation of rs10841847 genotypes and *CLEC4E* mRNA expression in additive **(E)** and dominant model **(F)** from the 1000 Genomes Project. The correlation of rs10841847 and *CLEC4E* mRNA expression in whole blood samples from the GTEx **(G)**. The correlation of rs11225211 and *BIRC3* mRNA expression levels in normal lung tissues from the GTEx **(H)**. PLCO, The Prostate, Lung, Colorectal and Ovarian Cancer Screening Trial; DSS, disease-specific survival; OS, overall survival; NSCLC, non-small cell lung cancer; ROC, receiver operating characteristic; GTEx, Genotype-Tissue Expression project.

To clarify collective effect of the two independent SNPs on NSCLC survival, we combined their protective alleles (i.e., *CLEC4E* rs10841847 A allele and *BIRC3* rs11225211 A allele) into one variable as a genetic score. NSCLC patients were categorized into four groups (i.e., 0, 1, 2, and 3-4) according to the number of their protective alleles (NPA). Similarly, an increased NPA was associated with better NSCLC OS and DSS in the PLCO dataset (*P*_trend_= 0.0002 and 0.002, respectively) after adjustment for available covariates (**Table 3**). Furthermore, we also dichotomized all NSCLC patients into two groups: 0-1 or 2-4 NPA. As shown in **Table 3**, compared with the 0-1 NPA group, the 2-4 group had a significantly favorable NSCLC OS and DSS in the PLCO dataset (*P*_trend_= 0.004 and 0.009, respectively). In addition, we further constructed KM survival curves to visualize the associations between NPA and NSCLC survival. As shown in **Figures 2A, B**, compared with the 2-4 NPA group, NSCLC KM survival curves of the 0-1 NPA group significantly declined (Log-rank *P*= 0.024 for DSS and Log-rank *P*= 0.027 for OS).

### Stratified Analysis for the Effect of NPA on NSCLC Survival

To evaluate whether the effect of NPA on NSCLC survival was confounded by other clinical covariates, we performed stratified analysis in the PLCO dataset. Compared with NSCLC patients having 0-1 NPA, those with 2-4 NPA had a significantly better NSCLC survival, except for the subgroup with age>71, female, former smoking status, late tumor stage, and clinical therapy. No significant interactions between protective alleles and each covariate on NSCLC survival were observed (**Supplementary Table S5**).

### Time-Dependent AUC and ROC Curves of the Two Independent SNPs for NSCLC Survival Prediction

To further evaluate predictive value of the two independent SNPs, time-dependent AUC and ROC curves were performed for NSCLC patients in the presence of available clinical covariates. In the PLCO trial, although the time-dependent AUC increased when protective alleles were added, the predictive performance of 60-month (5-year) NSCLC survival ROC curves was non-significantly improved (*P*=0.074 for DSS and *P*=0.070 for OS) (**Supplementary Figures S4B, D**). However, the predictive performance of 120-month (10-year) NSCLC survival ROC curves was significantly improved by adding protective alleles (*P*=0.028 for DSS and *P*=0.046 for OS) (**Figures 2C, D**).

### Bioinformatics Functional Prediction of the Two SNPs

To assess specific biological functions of the two independent SNPs associated with NSCLC survival, we explored SNP-related genomics data using an online bioinformatics tool (HaploReg, https://pubs.broadinstitute.org/mammals/haploreg/haploreg.php). It appears that *CLEC4E* rs10841847 is located in a DNase district, and a G>A change may be involved in modifying several protein motifs; *BIRC3* rs11225211 is predicted to be located in an enhancer histone marker district and a G>A change may be involved in disturbing several proteins’ expression (**Supplementary Table S6**). According to the data extracted from the Encyclopedia of DNA Elements (ENCODE) project, *CLEC4E* rs10841847 is probably located on the H3K4Me1 motif, while *BIRC3* rs11225211 is located in the region with enriched H3K4Me1 and H3K4Me3 as well as the possible binding site of Txn factor (**Supplementary Figure S5**). These findings imply a strong possibility that these two SNPs may modulate their gene expression levels through transcriptional regulation.

### Two Independent SNPs and Their Corresponding mRNA Expression

To further investigate molecular mechanisms underlying the associations between two identified SNPs and NSCLC survival, we explored correlations between protective alleles of two identified SNPs and their corresponding mRNA expression levels using eQTL analyses. In the RNA-Seq data on lymphoblastoid cell lines from 373 European descendants (obtained from the 1000 Genomes Project), the rs10841847 A allele showed a significant correlation with increased expression levels of *CLEC4E* mRNA in additive and dominant models (*P*=0.001 and *P*=0.0002, respectively) (**Figures 2E, F**), but not in recessive models (**Supplementary Figure S6A**). The rs11225211 A allele showed no correlation with *BIRC3* mRNA expression in any of additive, dominant, or recessive models (**Supplementary Figures S6B–D**). Moreover, we also performed the eQTL analysis by extracting data from the GTEx Project. The results showed that rs10841847 A allele was significantly associated with elevated *CLEC4E* mRNA expression levels in normal whole blood samples (*P*<0.0001) (**Figure 2G**), but not in normal lung tissues (**Supplementary Figure S6E**). The rs11225211 A allele was significantly correlated with lower *BIRC3* mRNA expression levels in normal lung tissues (*P*=0.027) (**Figure 2H**), but not in normal whole blood samples (**Supplementary Figure S6F**).

### *CLEC4E* and *BIRC3* Expression Levels and Lung Cancer Survival

Finally, we explored mRNA expression of *CLEC4E* and *BIRC3* in normal lung tissues and primary lung cancer tissues available from the TCGA database (data obtained from http://ualcan.path.uab.edu/index.html). As shown in (**Supplementary Figures S7A**–**D**), compared with normal lung tissues, mRNA expression levels of *CLEC4E* were significantly lower in both primary lung adenocarcinoma tissues and lung squamous cell carcinoma tissues; mRNA expression levels of *BIRC3* were significantly higher in primary lung adenocarcinoma tissues, but lower in lung squamous cell carcinoma tissues. Notably, as shown by the KM survival curve in **Supplementary Figures S7G, H**, lower mRNA expression levels of *BIRC3* were significantly associated with better survival of lung adenocarcinoma in two different online databases. However, mRNA expression levels of *CLEC4E* showed no association with NSCLC survival in these databases (**Supplementary Figures S7E**, **F**, **I**).

## Discussion

In the present study, we evaluated the associations between 18,588 SNPs of a set of 195 DAMPs-related pathway genes and NSCLC survival by using available genotyping and clinical outcome data from two previously published NSCLC GWAS datasets. Using this pathway approach, we identified two SNPs (i.e., *CLEC4E* rs10841847 G>A and *BIRC3* rs11225211 G>A) that were independently associated with NSCLC survival. In addition, we found that the rs10841847 A allele and rs11225211 A allele were associated with significantly higher *CLEC4E* (in peripheral blood lymphocytes from 1000 Genomes) and lower *BIRC3* mRNA expression (in normal lung tissues) levels, respectively. Moreover, we observed that levels of both *CLEC4E* and *BIRC3* mRNA expression were altered in NSCLC tissues and that a higher expression level of *BIRC3* mRNA was significantly associated with a poorer survival in NSCLC patients.

To date, there was no published report that described an association between genetic variants of *CLEC4E* and NSCLC survival. Because monitoring of the DAMPs-associated processes is difficult, we instead investigated SNPs in DAMPs-related pathway genes as independent prognostic biomarkers for NSCLC survival in a multivariate analysis. This multivariate model included adjustment for available covariates (i.e., age, sex, tumor stage, histology, smoking status, chemotherapy, radiotherapy, and surgery) as well as 28 previously published NSCLC survival-associated SNPs. As a result, we identified these two SNPs (i.e., rs1084147 and rs11225211) of the pathway genes, which collectively predicted prognosis of NSCLC patients.

*CLEC4E* is a C-type lectin domain family 4 member E (also known as *MINCLE*) that has been implicated in stimulating cell death-induced DAMPs, known to play a key role in antifungal and antibacterial immunity (41). It is known that CLEC receptors have potential regulatory effects on immune cell trafficking and modulatory effects on cancer cell activity in tumor microenvironment (TMB). But so far, there is no study about the role of CLEC4 as an immune regulator of TMB. Recently, it has been shown that *CLEC4E* is involved in enhancing the aggressiveness of urothelial cancer (42), and a new reported inhibitor of cancer cell invasion was identified based on its role in binding to the *CLEC4E* receptor (43). However, few studies have investigated the roles of *CLEC4E* in NSCLC. To our knowledge, the present study is the first to report an association between genetic variants of *CLEC4E* and NSCLC survival. Notably, the *CLEC4E* rs10841847 G>A showed a significant protective effect on NSCLC survival and a significant association with increased *CLEC4E* mRNA expression levels in both normal lymphoblastoid cells and whole blood cells. Moreover, *CLEC4E* mRNA expression was much more weakened in NSCLC tissues than in normal lung tissues. These observations imply that *CLEC4E* may function as a suppressor gene in NSCLC but additional functional studies are needed to support this speculation.

*BIRC3* (also known as baculoviral IAP repeat containing 3) encodes the protein c-IAP2, an inhibitor of an apoptosis-associated proteins family. *BIRC3* has been shown to regulate the molecular signaling cell apoptosis, inflammatory signaling, cell proliferation and migration (44, 45). Previous studies have revealed that accumulated *BIRC3* contributes to tumor progression in several malignancies (46, 47), and the expression levels of *BIRC3* have been shown to be correlated with prognosis of patients with different cancers (48, 49). In the present study, we evaluated associations between genetic variants of *BIRC3* and NSCLC survival and found that the *BIRC3* rs11225211 G>A had a significant protective effect on NSCLC survival. Interestingly, we also found that *BIRC3* mRNA expression was accumulated conspicuously in lung adenocarcinoma tissues but weakened in lung squamous cell carcinoma tissues in the TCGA dataset. Additional studies are needed to explore if BIRC3 may serve as a potential biomarker of lung adenocarcinoma. Furthermore, the lower BIRC3 mRNA expression was obviously associated with a better survival in patients with lung cancer in the Human Protein Atlas database. A previous study suggested that BIRC3 might play a role of tumor suppressor, because its deficiency was associated with poor prognosis of the patients (50), which is consistent with our data. Taken together, these results suggest that BIRC3 may function as a suppressor gene in NSCLC, but this speculation also needs to be substantiated in additional molecular biology experiments and clinical studies in the future.

Despite the above-mentioned significant observations, there are several limitations in the present study. First, NSCLC patients in both discovery and validation datasets were recruited only from Caucasian populations, further validation in other NSCLC patient cohorts of different ethnicities should be pursued. Additionally, the PLCO discovery and HLCS replication datasets had differences in the distributions of baseline characteristics, which may partially influence the replication results, leading to fewer significant SNPs being identified. Finally, the sample sizes of these two datasets were still not large enough to perform the FDR test for multiple comparison correction; however, considering that nearly 91% of selected SNPs were imputed in the present study, the BFDP test might be more appropriate for these highly correlated SNPs under investigation.

## Data Availability Statement

The datasets presented in this study can be found in online repositories. The names of the repository/repositories and accession number(s) can be found in the article/**Supplementary Material**.

## Author Contributions

Conception and design: QW, LHL, and DC. Development of methodology: QW, HL, and DC. Acquisition of data (provided animals, acquired and managed patients, provided facilities, etc.): QW, HL, and DC. Analysis and interpretation of data (e.g., statistical analysis, biostatistics, computational analysis): QW, HL, LHL, and SL. Writing, review, and/or revision of the manuscript: All authors. Administrative, technical, or material support (i.e., reporting or organizing data, constructing databases): QW, HL, LS, LJL, and DC. Study supervision: QW, HL, and DC. All authors contributed to the article and approved the submitted version.

## Funding

This work was supported by the National Institute of Health [CA090578, CA074386, CA092824, U01CA209414]; the Duke Cancer institute as part of the P30 Cancer Center Support Grant [NIH/NCI CA014236]; and the V Foundation for Cancer Research [D2017-19]; The National Natural Science Foundation of China (81760419);2018 Guangxi One Thousand Young and Middle-Aged College and University Backbone Teachers Cultivation Program to LHL, P.R. China; “Medical Excellence Award” Funded by the Creative Research Development Grant from the First Affiliated Hospital of Guangxi Medical University to LHL.

## Author Disclaimer

The funders had no involvement in the study design, the collection, analysis, and interpretation of data, the writing of this report, and the decision to submit for publication.

## Conflict of Interest

The authors declare that the research was conducted in the absence of any commercial or financial relationships that could be construed as a potential conflict of interest.

## Publisher’s Note

All claims expressed in this article are solely those of the authors and do not necessarily represent those of their affiliated organizations, or those of the publisher, the editors and the reviewers. Any product that may be evaluated in this article, or claim that may be made by its manufacturer, is not guaranteed or endorsed by the publisher.

## Abbreviations

SNPs, single nucleotide polymorphisms; NSCLC, Non-small cell lung cancer; DAMPs, Damage-associated molecular patterns; LUAD, lung adenocarcinoma; LUSC: lung squamous cell carcinoma; GWAS, Genome-Wide Association Study; *BIRC3*, baculoviral IAP repeat containing 3; *CLEC4E*,C-type lectin domain family 4 member E;PLCO, the Prostate, Lung, Colorectal and Ovarian Cancer Screening Trial; HLCS, Harvard Lung Cancer Susceptibility; OS, overall survival; DSS: disease-special survival; LD, linkage disequilibrium; FDR, false discovery rate; BFDP, Bayesian false discovery probability; eQTL, expression quantitative trait loci; TCGA, the Cancer Genome Atlas; ROC, receiver operating characteristic; EAF, effect allele frequency; HR, hazards ratio; CI: confidence interval; AUC, area under the receiver operating characteristic curve; GTEx, genotype-tissue expression project; NPA, number of protective alleles.
