# Supplementary material for: Genetic Variants of CLEC4E and BIRC3 in Damage-Associated Molecular Patterns-Related Pathway Genes Predict Non-Small Cell Lung Cancer Survival
Source: Front Oncol. 2021 Oct 6;11:717109. doi: 10.3389/fonc.2021.717109 (PMC8527850; doi:10.3389/fonc.2021.717109)
Supplement: Supplementary file 1 [file DataSheet_1.docx]

**Supplementary Table S1. Comparison of the characteristics between the PLCO trial and the HLCS study**

| **Characteristics** | **PLCO** | | **HLCS** | | ***P*^*^** |
| --- | --- | --- | --- | --- | --- |
|  | **Frequency** | **Deaths (%)** | **Frequency** | **Deaths (%)** |  |
| Total | 1185 | 798 (67.3) | 984 | 665 (67.5) |  |
| Median overall survival (months) | 23.8 |  | 39.9 |  |  |
| Age |  |  |  |  |  |
| ≤71 | 636 | 400 (62.9) | 654 | 428 (65.4) | <0.0001 |
| >71 | 549 | 398 (72.5) | 330 | 237 (71.8) |  |
| Sex |  |  |  |  |  |
| Male | 698 | 507 (72.6) | 507 | 379 (74.7) | 0.0006 |
| Female | 487 | 291 (59.8) | 477 | 286 (59.9) |  |
| Smoking status |  |  |  |  |  |
| Never | 115 | 63 (54.8) | 92 | 52 (56.5) | 0.166 |
| Current | 423 | 272 (64.3) | 390 | 266 (68.2) |  |
| Former | 647 | 463 (71.6) | 502 | 347 (69.1) |  |
| Histology |  |  |  |  |  |
| Adenocarcinoma | 577 | 348 (60.3) | 597 | 378 (63.3) | <0.0001 |
| Squamous cell carcinoma | 285 | 192 (67.4) | 216 | 156 (72.2) |  |
| Others | 323 | 258 (79.9) | 171 | 131 (76.6) |  |
| Stage |  |  |  |  |  |
| I - IIIA | 655 | 315 (48.1) | 606 | 352 (58.0) | 0.003 |
| IIIB - IV | 528 | 482 (91.3) | 377 | 313 (83.0) |  |
| *Missing* | 2 |  | -- |  |  |

Abbreviations: PLCO, the Prostate, Lung, Colorectal and Ovarian Cancer Screening Trial; HLCS, Harvard Lung Cancer Suceptibility Study.

***** Chi-square test for the comparison of the characteristics between the PLCO trial and Harvard study for each clinical variable.

| **Supplementary Table S2. List of 195 selected genes in the damage-associated molecular pattern-related gene-set used in the discovery analysis** | | | | |
| --- | --- | --- | --- | --- |
| **Dataset** | **Name of pathway** | **Selected genes^a^** | **Number of genes** |  |
| GO | CYTOPLASMIC_PATTERN_RECOGNITION_RECEPTOR_SIGNALING_PATHWAY | *ALPK1, ANKRD17, BIRC2, BIRC3, C1QBP, CASP8, CYLD, DDX58, DDX60, DHX58, ERBIN, GPATCH3, HSPA1A, HSPA1B, IFIH1, IKBKG, INAVA, IRAK1, IRAK2IRF3, IRF7, IRGM, ITCH, LSM14A, MAP2K6, MAP3K7, NFKBIA, NLRX1, NOD1 NOD2, NOP53, NPLOC4, OTULIN, PTPN22, PUM1, PUM2, RELA, RIOK3, RIPK2, RNF125, RPS27A, SEC14L1, TAB1, TAB2, TAB3, TIFA, TKFC, TLR4, TMEM173, TNFAIP3, TRAF6, TRIM15, TSPAN6, UBA52, UBB, UBC, UBE2N, UBE2V1, UFD1, USP17L2, XIAP, ZCCHC3* | 62 |  |
| GO | PATTERN_RECOGNITION_RECEPTOR_ACTIVITY | *CD14, CD36, CLEC4E, CLEC7A, COLEC12, DMBT1, FCN1, LY96, MARCO, PGLYRP1, PGLYRP2, PGLYRP3, PGLYRP4, PTAFR, SCARB1, TLR2, TLR4, TLR7, TLR8, TLR9, TRIM5* | 21 |  |
| GO | PATTERN_RECOGNITION_RECEPTOR_SIGNALING_PATHWAY | *ACOD1, ALPK1, ANKRD17, APOB, APPL1, APPL2, ARRB2, BCL10, , BIRC2, BIRC3, BPIFB1, BTK, C1QBP, CACTIN, CASP8, CAV1, CD14, CD300A, CD300LF, CD36, CHUK, CLEC4E, CLEC7A, CNPY3, COLEC12, CTSB, CTSK, CTSL, CTSS, CYBA, CYLD, DAB2IP, DDX58, DDX60, DHX58, DMBT1, ERBIN, ESR1, F2RL1, FADD, FCN1, FCRL3, FFAR2, FGA, FGB, FGG, FLOT1, GFI1, GPATCH3, GPS2, GRAMD4, HAVCR2, HMGB1, HSP90B1, HSPA1A, HSPA1B, HSPD1, IFIH1, IKBKB, IKBKE, IKBKG, INAVA, IRAK1, IRAK2, IRAK3, IRAK4, IRF1, IRF3, IRF4, IRF7, IRGM, ITCH, ITGAM, ITGB2, LBP, LGMN, LGR4, LILRA2, LILRA4, LRRC14, LSM14A, LTF, LY96, LYN, MAP2K6, MAP3K1, MAP3K7, MAPKAPK2, MAPKAPK3, MARCO, MFHAS1, MIR146A, MIR146B, MIR17, MIR19A, MYD88, NFKBIA, NFKBIL1, NLRP2B, NLRP6, NLRX1, NOD1, NOD2, NOP53, NPLOC4, NR1D1, NR1H3, NR1H4, OTUD4, OTULIN, PDPK1, PELI1, PGLYRP1, PGLYRP2, PGLYRP3, PGLYRP4, PIK3AP1, PIK3C3, PIK3R4, PJA2, PRKCE, PTPN22, PTPRS, PUM1, PUM2, RAB7B, REG3G, RELA, RFTN1, RIOK3, RIPK1, RIPK2, RNF125, RPS27A, RPS6KA3, RSAD2, RTN4, S100A1, S100A14, S100A8, S100A9, SARM1, SEC14L1, SFTPA1, SFTPA2, SMPDL3B, TAB1, TAB2, TAB3, TANK, TBK1, TICAM1, TICAM2, TIFA, TIRAP, TKFC, TLR1, TLR10, TLR2, TLR3, TLR4, TLR5, TLR6, TLR7, TLR8, TLR9, TMEM173, TNFAIP3, TNIP1, TNIP2, TNIP3, TRAF3, TRAF6, TREML4, TRIL, TRIM15, TRIM5, TSPAN6, TYRO3, UBA52, UBB, UBC, UBE2D1, UBE2D2, UBE2D3, UBE2N, UBE2V1, UBQLN1, UFD1, UNC93B1, USP17L2, WDFY1, XIAP, ZCCHC3* | 195 |  |
| REACTOME | - | *-* | 0 |  |
| BIOCARTA | - | *-* | 0 |  |
| PID | - | *-* | 0 |  |
| KEGG | - | *-* | 0 |  |
| Total |  | *GPATCH3, INAVA, PTPN22, PUM1, PGLYRP3, PGLYRP4, PTAFR, BCL10, CTSK, CTSS, FCRL3, GFI1, IKBKE, MAPKAPK2, S100A1, S100A14, S100A8, S100A9, SMPDL3B, TLR5, CASP8, IFIH1, PUM2, RPS27A, MARCO, APOB, HSPD1, PELI1, PRKCE, REG3G, RSAD2, RTN4, TANK, WDFY1, IRAK2, TLR9, APPL1, LTF, MAPKAPK3, MYD88, PIK3R4, RFTN1, ALPK1, ANKRD17, DDX60, TIFA, TLR2 FGA, FGB, FGG, OTUD, TLR1, TLR10, TLR3, TLR6, TNIP2, TNIP3, UBE2D3, ERBIN, IRGM, OTULIN, TMEM173, CD14, F2RL1, HAVCR2, IRF1, MAP3K1, MIR146A, PJA2, TICAM2, TNIP1, UBE2D2, HSPA1A, HSPA1B, MAP3K7, TAB2, TNFAIP3, TRIM15, CNPY3, ESR1, FLOT1, IRF4, NFKBIL1, RIPK1, TREML4, NOD1, CD36, CAV1, TRIL, RIPK2, USP17L2, LY96, CTSB, IKBKB, LRRC14, LYN, MFHAS1, DDX58, TLR4, FCN1, CTSL, DAB2IP, UBQLN1, DMBT1, CHUK, MIR146B, PIK3AP1, SFTPA1, SFTPA2, UBE2D1, BIRC2, BIRC3, IRF7, NLRX1, RELA, TKFC, TRAF6, TRIM5, FADD, LGR4, NLRP6, NR1H3, TIRAP, UNC93B1, UBC, UBE2N, CLEC4E, CLEC7A, SCARB1, APPL2, HSP90B1, IRAK3, IRAK4, NR1H4, TBK1, ACOD1, HMGB1, MIR17, MIR19A, NFKBIA, LGMN, TRAF3, TYRO3, CYLD, NOD2, CYBA, ITGAM, PDPK1, C1QBP, DHX58, MAP2K6, NPLOC4, SEC14L1, UBB, ARRB2, CD300A, CD300LF, GPS2, NR1D1, SARM1, RIOK3, RNF125, COLEC12, PIK3C3, IRF3, LSM14A, NOP53, UBA52, PGLYRP1, PGLYRP2, CACTIN, FFAR2, LILRA2, LILRA4, PTPRS, TICAM1, ITCH, UBE2V1, ZCCHC3, BPIFB1, LBP, ITGB2, TAB1, UFD1, GRAMD4, IKBKG, IRAK1, TAB3, TSPAN6, XIAP, TLR7, TLR8, BTK, NLRP2B, RPS6KA3* | 195^b^ |  |
| ^a^ Genes were selected based on online datasets (<http://software.broadinstitute.org/gsea/msigdb/search.jsp>) and literatures.  ^b^ 83 duplicated genes had been removed.  Keyword: damage-associated AND molecular AND pattern.  Organism: Homo sapiens. | | | |  |

**Supplementary Table S3b. Associations of nine validated significant SNPs with overall survival in both discovery and validation datasets from two previously published NSCLC GWAS datasets**

|  | | **PLCO (n=1185)** | | | **HLCS (n=984) Combined-analysis** |
| --- | --- | --- | --- | --- | --- |
| **SNP** | **Allelea** | **Gene** |  |  | |

| **rs10841847** | **G>A** | ***CLEC4E*** | **0.61** | **0.792** | **0.438** | **0.88 (0.79-0.97)** | **0.012** |  | **0.89 (0.80-0.99)** | **0.039** | **0.87** | **0** | **0.89 (0.82-0.95)** | **1.41x10^-3^** |
| --- | --- | --- | --- | --- | --- | --- | --- | --- | --- | --- | --- | --- | --- | --- |
| rs10841856 | T>C | *CLEC4E* | 0.61 | 0.792 | 0.436 | 0.88 (0.79-0.97) | 0.012 |  | 0.89 (0.80-0.99) | 0.040 | 0.88 | 0 | 0.88 (0.82-0.95) | 0.001 |
| **rs11225211** | **G>A** | ***BIRC3*** | **0.61** | **0.687** | **0.170** | **0.82 (0.72-0.95)** | **0.006** |  | **0.81 (0.68-0.98)** | **0.029** | **0.94** | **0** | **0.82 (0.73-0.91)** | **3.75x10^-4^** |
| rs1711114 | G>A | *IRAK2* | 0.61 | 0.469 | 0.147 | 0.81 (0.70-0.93) | 0.003 |  | 0.86 (0.73-1.00) | 0.580 | 0.58 | 0 | 0.83 (0.75-0.92) | 6x10^-4^ |
| rs3788142 | G>A | *ITGB2* | 0.61 | 0.768 | 0.241 | 1.17 (1.04-1.32) | 0.008 |  | 1.15 (1.01-1.32) | 0.030 | 0.85 | 0 | 1.16 (1.06-1.27) | 0.001 |
| rs3942261 | G>A | *APPL1* | 0.61 | 0.499 | 0.089 | 0.77 (0.64-0.92) | 0.005 |  | 0.75 (0.61-0.92) | 0.007 | 0.85 | 0 | 0.76 (0.66-0.87) | 8.21x10^-5^ |
| rs7307228 | C>T | *CLEC4E* | 0.61 | 0.792 | 0.438 | 0.88 (0.79-0.97) | 0.011 |  | 0.89 (0.80-0.99) | 0.039 | 0.88 | 0 | 0.88 (0.82-0.95) | 0.001 |
| rs779901 | C>T | *IRAK2* | 0.61 | 0.327 | 0.141 | 0.78 (0.67-0.91) | 0.001 |  | 0.84 (0.72-0.98) | 0.031 | 0.5 | 0 | 0.81 (0.73-0.90) | 1x10^-4^ |
| rs779903 | G>A | *IRAK2* | 0.61 | 0.327 | 0.141 | 0.78 (0.67-0.91) | 0.001 |  | 0.84 (0.78-0.98) | 0.032 | 0.45 | 0 | 0.78 (0.67-0.91 | 1.7x10^-5^ |

**FDRa BFDPa MAF HR (95% CI)b *P* b HR (95% CI)c *P* c *P*het d *I* 2 HR (95% CI)b *P*e**

**t**

Abbreviations: SNP, single nucleotide polymorphism; NSCLC, non-small cell lung cancer; GWAS, genome-wide association study; PLCO, Prostate, Lung, Colorectal and Ovarian cancer screening trial; HLCS: Harvard Lung Cancer Susceptibility; MAF, minor allele frequency; HR, hazard ratio; CI, confidence interval; FDR: false discovery rate; BFDP: Bayesian false discovery probability

aFDR and BFDP were available in the PLCO dataset because the HLCS study provided only the summary data

bObtained from an additive genetic model with adjustment for age, sex, stage, histology, smoking status, chemotherapy, radiotherapy, surgery, PC1, PC2, PC3, and PC4; cObtained from an additive genetic model with adjustment for age, sex, stage, histology, smoking status, chemotherapy, radiotherapy, surgery, PC1, PC2, and PC3; d*P***het**: *P* value for heterogeneity by Cochrane’s Q test;

eMeta-analysis in the fixed-effects model.

| **Supplementary Table S4. Associations of the first 10 principal components and OS of NSCLC in the PLCO trial** | | | | |
| --- | --- | --- | --- | --- |
| **PC*** | **Parameter Estimate** | **Standard Error** | **Chi-Square** | ***P*** |
| **PC1** | **4.821** | **1.353** | **12.697** | **<0.001** |
| **PC2** | **-0.681** | **1.228** | **0.308** | **0.579** |
| **PC3** | **-3.054** | **0.949** | **10.351** | **0.001** |
| **PC4** | **-2.837** | **1.246** | **5.184** | **0.023** |
| PC5 | -0.910 | 1.232 | 0.546 | 0.460 |
| PC6 | 1.355 | 1.252 | 1.172 | 0.279 |
| PC7 | -0.236 | 1.218 | 0.038 | 0.846 |
| PC8 | -1.684 | 1.322 | 1.622 | 0.203 |
| PC9 | -1.886 | 1.267 | 2.216 | 0.137 |
| PC10 | 0.347 | 1.240 | 0.078 | 0.180 |
| Abbreviations: OS, overall survival; NSCLC, non-small cell lung cancer; PLCO, the Prostate, Lung, Colorectal and Ovarian Cancer Screening Trial; PC, principal component.  ***** The first 4 PC were used for the adjustment for population stratification in the multivariate analysis. | | | | |

**Supplementary Table S5. Stratified analysis for associations between the protective alleles and survival of NSCLC in the PLCO trial**

| **Characteristics** | | **0-1 protective**  **alleles** | **2-4 protective**  **alleles** | | | **Multivariate Analysis^b^ for OS** | | | **Multivariate Analysis^b^ for DSS** | | | |
| --- | --- | --- | --- | --- | --- | --- | --- | --- | --- | --- | --- | --- |
|  |  | **Frequency^a^** | **Frequency^a^** |  |  | **HR (95% CI)** | ***P*** | ***P* _inter_^c^** | **HR (95% CI)** | ***P*** | ***P* _inter_^c^** |  |
| Age (years) | |  |  |  |  |  |  |  |  |  |  |  |
| ≤ 71 | | 426 | 208 |  |  | 0.80 (0.64-0.99) | 0.040 |  | 0.76 (0.61-0.96) | 0.023 |  |  |
| > 71 | | 339 | 201 |  |  | 0.86 (0.69-1.06) | 0.163 | 0.958 | 0.88 (0.70-1.10) | 0.249 | 0.704 |  |
| Sex | |  |  |  |  |  |  |  |  |  |  |  |
| Male | | 440 | 255 |  |  | 0.75 (0.62-0.90) | 0.002 |  | 0.77 (0.63-0.94) | 0.012 |  |  |
| Female | | 325 | 154 |  |  | 0.93 (0.72-1.20) | 0.562 | 0.131 | 0.88 (0.67-1.15) | 0.348 | 0.334 |  |
| Smoking status |  | |  |  |  |  |  |  |  |  |  |  |
| Never | | 69 | 45 |  |  | 1.53 (0.87-2.68) | 0.142 |  | 1.53 (0.88-2.68) | 0.134 |  |  |
| Current | | 289 | 128 |  |  | 0.80 (0.61-1.06) | 0.127 |  | 0.85 (0.63-1.14) | 0.266 |  |  |
| Former | | 407 | 236 |  |  | 0.75 (0.62-0.91) | 0.004 | 0.128 | 0.73 (0.59-0.90) | 0.003 | 0.063 |  |
| Histology | |  |  |  |  |  |  |  |  |  |  |  |
| Adeno | | 364 | 211 |  |  | 0.79 (0.62-0.99) | 0.038 |  | 0.80 (0.63-1.02) | 0.067 |  |  |
| Squamous | | 195 | 89 |  |  | 0.70 (0.51-0.97) | 0.030 |  | 0.72 (0.51-1.03) | 0.068 |  |  |
| Others | | 206 | 109 |  |  | 0.89 (0.67-1.67) | 0.039 | 0.138 | 0.86 (0.64-1.14) | 0.292 | 0.467 |  |
| Tumor stage | |  |  |  |  |  |  |  |  |  |  |  |
| I-IIIA | | 431 | 223 |  |  | 0.83 (0.65-1.05) | 0.122 |  | 0.84 (0.65-1.10) | 0.215 |  |  |
| IIIB-IV | | 334 | 186 |  |  | 0.86 (0.71-1.04) | 0.123 | 0.579 | 0.84 (0.69-1.03) | 0.088 | 0.591 |  |
| Chemotherapy | |  |  |  |  |  |  |  |  |  |  |  |
| No | | 417 | 220 |  |  | 0.74 (0.58-0.93) | 0.009 |  | 0.75 (0.58-0.96) | 0.023 |  |  |
| Yes | | 348 | 189 |  |  | 0.95 (0.78-1.17) | 0.628 | 0.139 | 0.92 (0.74-1.13) | 0.406 | 0.146 |  |
| Radiotherapy | |  |  |  |  |  |  |  |  |  |  |  |
| No | | 495 | 265 |  |  | 0.91 (0.74-1.11) | 0.350 |  | 0.91 (0.73-1.12) | 0.371 |  |  |
| Yes | | 270 | 144 |  |  | 0.73 (0.57-0.92) | 0.008 | 0.189 | 0.72 (0.57-0.92) | 0.009 | 0.218 |  |
| Surgery | |  |  |  |  |  |  |  |  |  |  |  |
| No | | 420 | 214 |  |  | 0.85 (0.71-1.02) | 0.074 |  | 0.85 (0.71-1.02) | 0.085 |  |  |
| Yes | | 345 | 195 |  |  | 0.91 (0.69-1.21) | 0.514 | 0.385 | 0.90 (0.65-1.23) | 0.507 | 0.560 |  |

Abbreviations: OS, overall survival; DSS, disease-specific survival; NSCLC, non-small cell lung cancer; PLCO, the Prostate, Lung, Colorectal and Ovarian Cancer Screening Trial; HR, hazards ratio; CI, confidence interval.

^a^ 11 missing date were excluded.

^b^ Adjusted for age, sex, stage, histology, smoking status, chemotherapy, radiotherapy, surgery, PC1, PC2, PC3, and PC4.

^c^ *P* _inter_: *P* value for interaction analysis between characteristic and protective alleles.

**Supplementary Table S6. Function prediction for *CLEC4E*rs10841847 and *BIRC3* rs11225211**

| **SNP** | **Gene** | | **Chr** | **Genotyped** | **Haploreg v4.1^a^** | | | | | |  |
| --- | --- | --- | --- | --- | --- | --- | --- | --- | --- | --- | --- |
|  |  |  |  |  | **Promoter histone marks** | **Enhancer histone marks** | **DNase** | **Motifs changed** | **Selected eQTL hits** |  |  |
| rs10841847 | | *CLEC4E* | 12 | no | -- | -- | THYM | 4 altered motifs | 11 hits |  |  |
| rs11225211 | *BIRC3* | | 11 | no | -- | BLD | -- | 4 altered motifs | 2 hits |  |  |
| Abbreviations: SNP, single nucleotide polymorphism; Chr, chromosome; DNase, deoxyribonuclease; eQTL, expression quantitative trait loci.  ^a^ Haploreg: <https://pubs.broadinstitute.org/mammals/haploreg/haploreg.php> | | | | | | | | | | | |

**Supplemental Acknowledgements**

We wish to thank all of the investigators and funding agencies that enabled the deposition of data in dbGaP and PLCO that we used in this study:

The datasets used for the analyses described in this manuscript were obtained from dbGaP at http://www.ncbi.nlm.nih.gov/gap through dbGaP accession number phs000336.v1.p1 and phs000093.v2.p2. Principal Investigators: Maria Teresa Landi. Genetic Epidemiology Branch, Division of Cancer Epidemiology and Genetics, National Cancer Institute, National Institutes of Health, Bethesda, MD, USA. Neil E. Caporaso. Genetic Epidemiology Branch, Division of Cancer Epidemiology and Genetics, National Cancer Institute, National Institutes of Health, Bethesda, MD, USA.

Funding support for the GWAS of Lung Cancer and Smoking was provided through the NIH Genes, Environment and Health Initiative [GEI] (Z01 CP 010200). The human subjects participating in the GWAS derive from The Environment and Genetics in Lung Cancer Etiology (EAGLE) case-control study and the Prostate, Lung Colon and Ovary Screening Trial and these studies are supported by intramural resources of the National Cancer Institute. Assistance with phenotype harmonization and genotype cleaning, as well as with general study coordination, was provided by the Gene Environment Association Studies, GENEVA Coordinating Center (U01HG004446). Assistance with data cleaning was provided by the National Center for Biotechnology Information. Funding support for genotyping, which was performed at the Johns Hopkins University Center for Inherited Disease Research, was provided by the NIH GEI (U01HG004438).

PLCO was also supported by the Intramural Research Program of the Division of Cancer Epidemiology and Genetics and by contracts from the Division of Cancer Prevention, National Cancer Institute, NIH, DHHS. The authors thank PLCO screening center investigators and staff, and the staff of Information Management Services Inc. and Westat Inc. Most importantly, we acknowledge trial participants for their contributions that made this study possible.
