# Supplementary material for: Genetic Variants of CLEC4E and BIRC3 in Damage-Associated Molecular Patterns-Related Pathway Genes Predict Non-Small Cell Lung Cancer Survival
Source: Front Oncol. 2021 Oct 6;11:717109. doi: 10.3389/fonc.2021.717109 (PMC8527850; doi:10.3389/fonc.2021.717109)
Supplement: Supplementary file 2 [file DataSheet_2.docx]

Info score ≥ 0.8

**Supplementary Figure S1. The distribution of the imputation info score in the present study.**

**
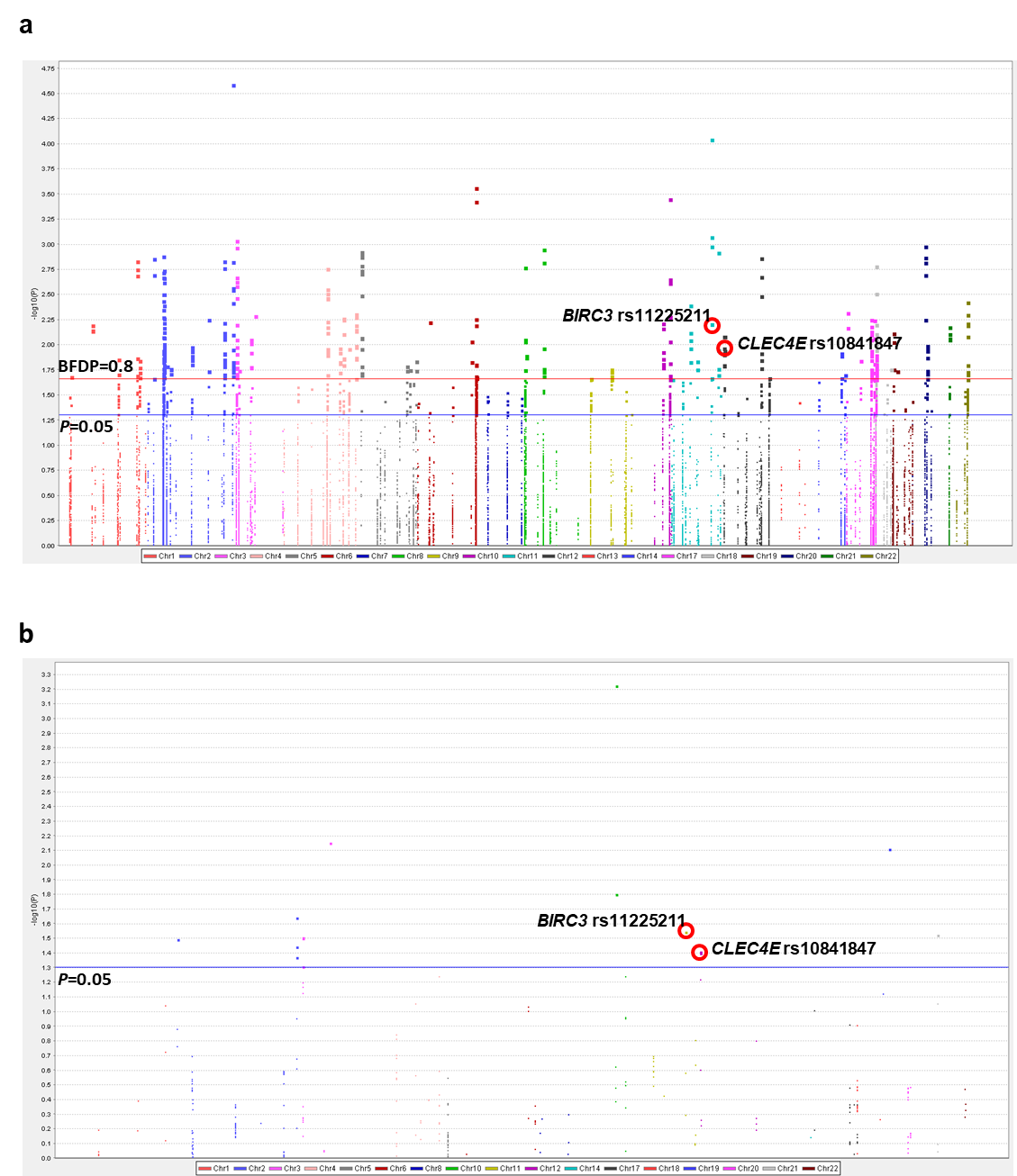
**

**Supplementary Figure S2. Manhattan plot.** Manhattan plot for 18,588 SNPs of DMAP-related pathway genes in the PLCO trial (a). Manhattan plot for 340 SNPs of DMAP-related pathway genes in the HLCS trial (b). The blue horizontal line indicates *P*=0.05 and the red line indicates BFDP=0.80. Abbreviations: DMAP, damage-associated molecular pattern; NSCLC, non-small cell lung cancer; PLCO, Prostate, Lung, Colorectal and Ovarian Cancer Screening Trial; BFDP, Bayesian false-discovery probability.

**Supplementary Figure S3. Regional association plots for two independent SNPs in the DMAP-related pathway genes.** Regional association plots contained 100 kb up or downstream of *CLEC4E* (a) and *BIRC3* (b). Data points are colored according to the level of LD of each pair of SNPs based on the hg19/1000 Genomes European population. The left-hand y-axis shows the association P-value of individual SNPs in the discovery dataset, which is plotted as -log10 (*P*) against chromosomal base-pair position. The right-hand y-axis shows the recombination rate estimated from HapMap Data Rel 22/phase II European population. The Regional association plots were generated using Locus Zoom (<http://locuszoom.org/>). Abbreviations: SNPs, single-nucleotide polymorphisms; DMAP, damage-associated molecular pattern; LD, linkage disequilibrium.


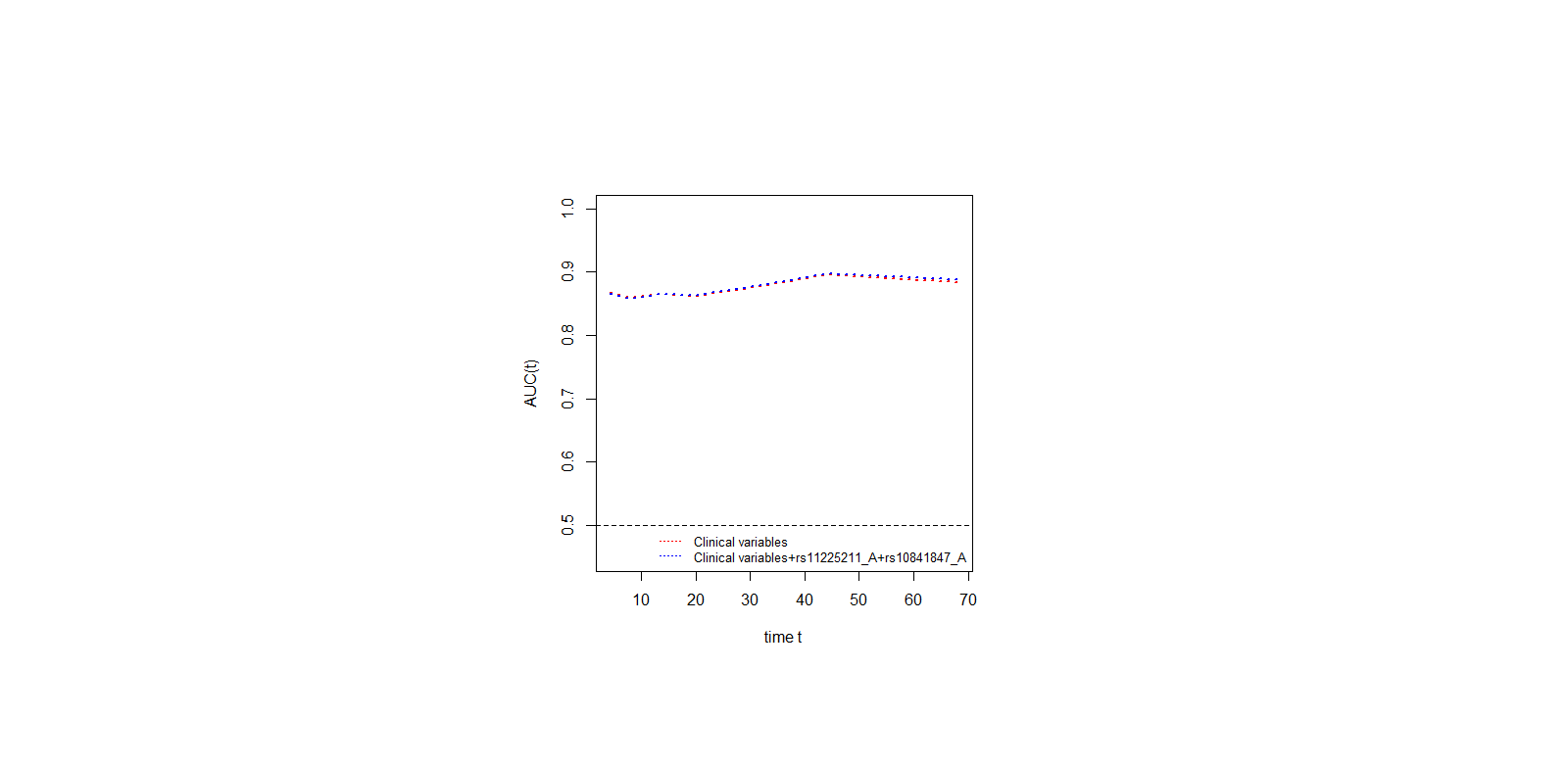

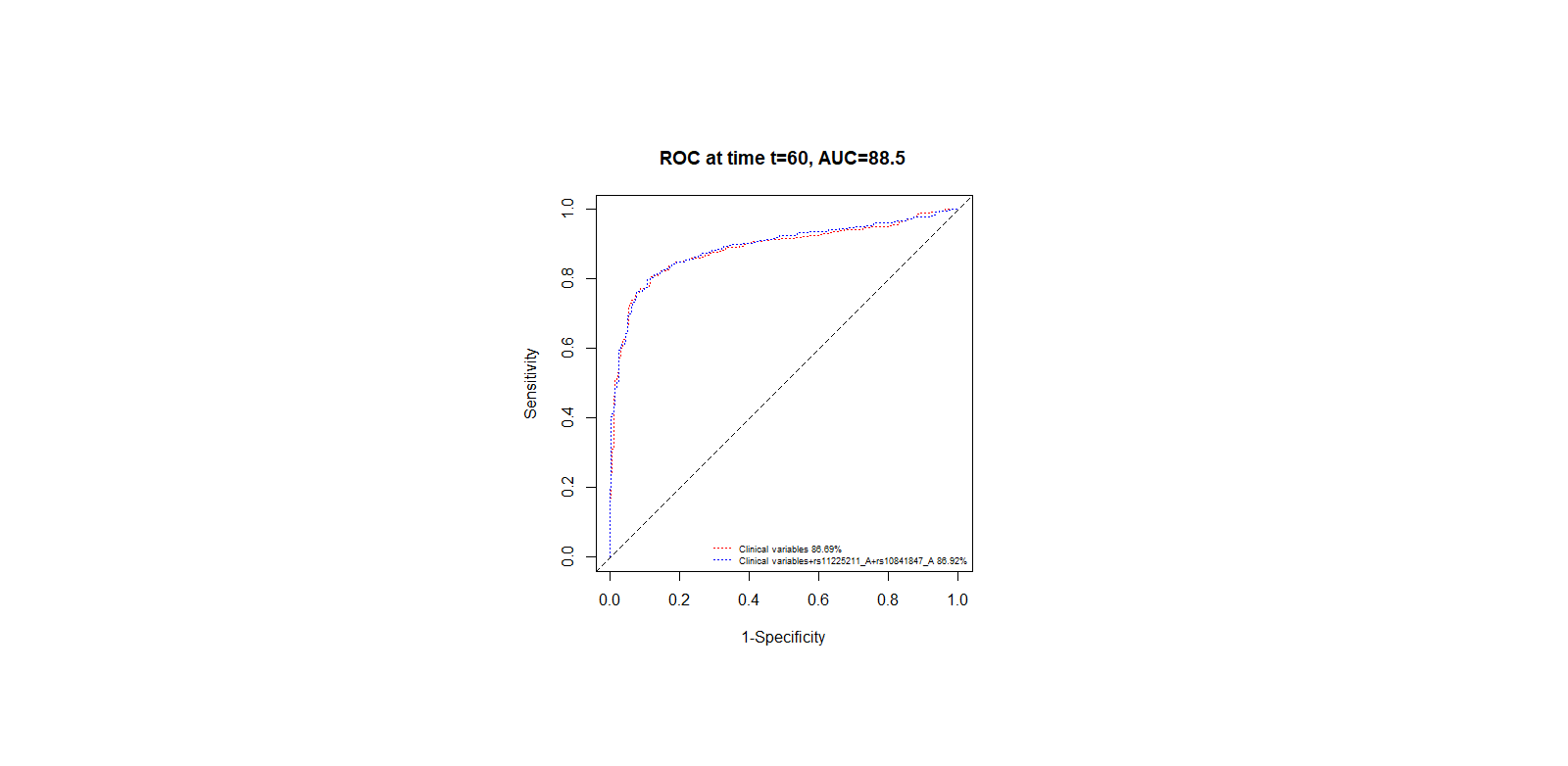


**time t**

**AUC(t)**

**Specificity**

**Sensitivity**


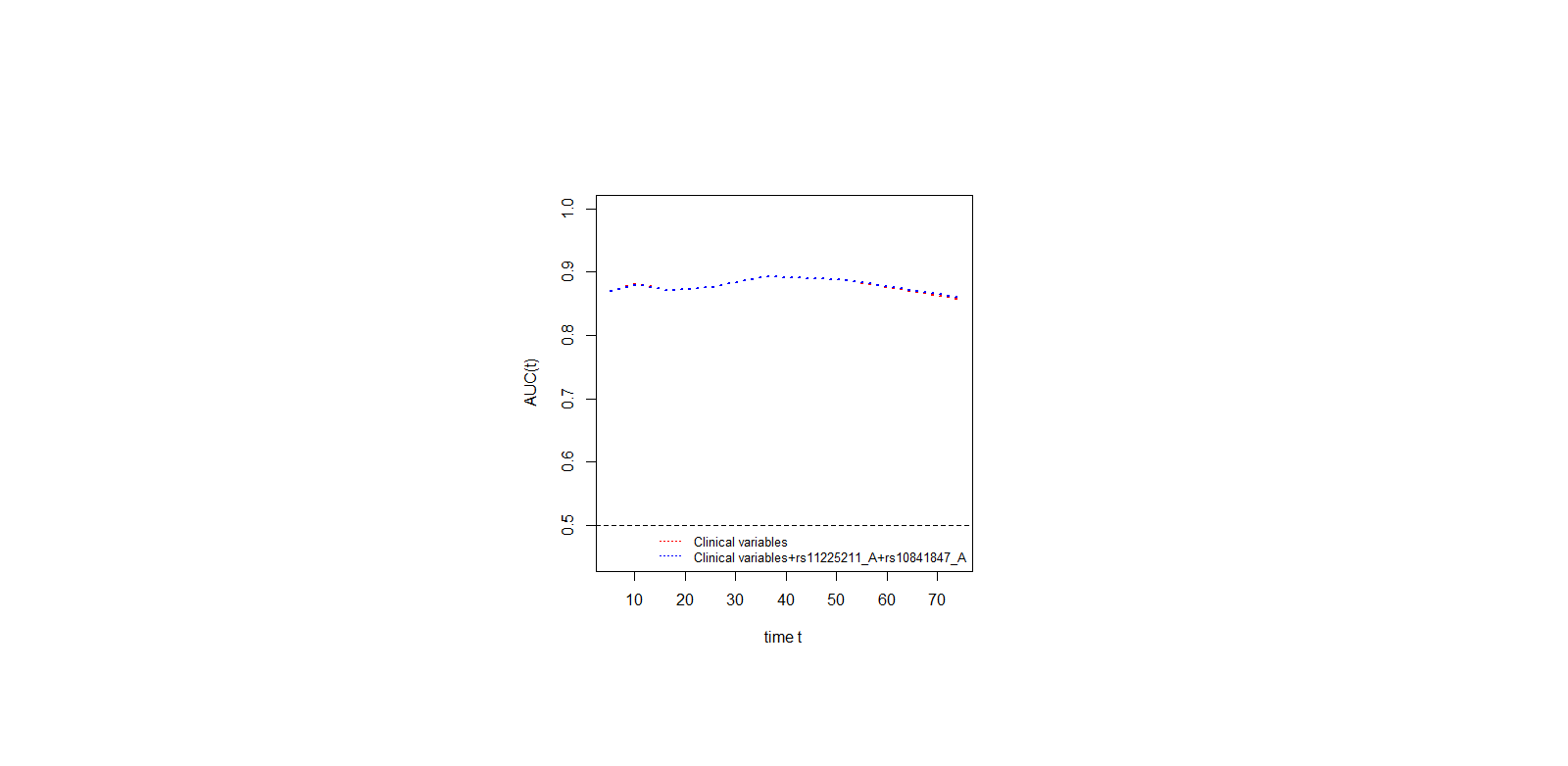

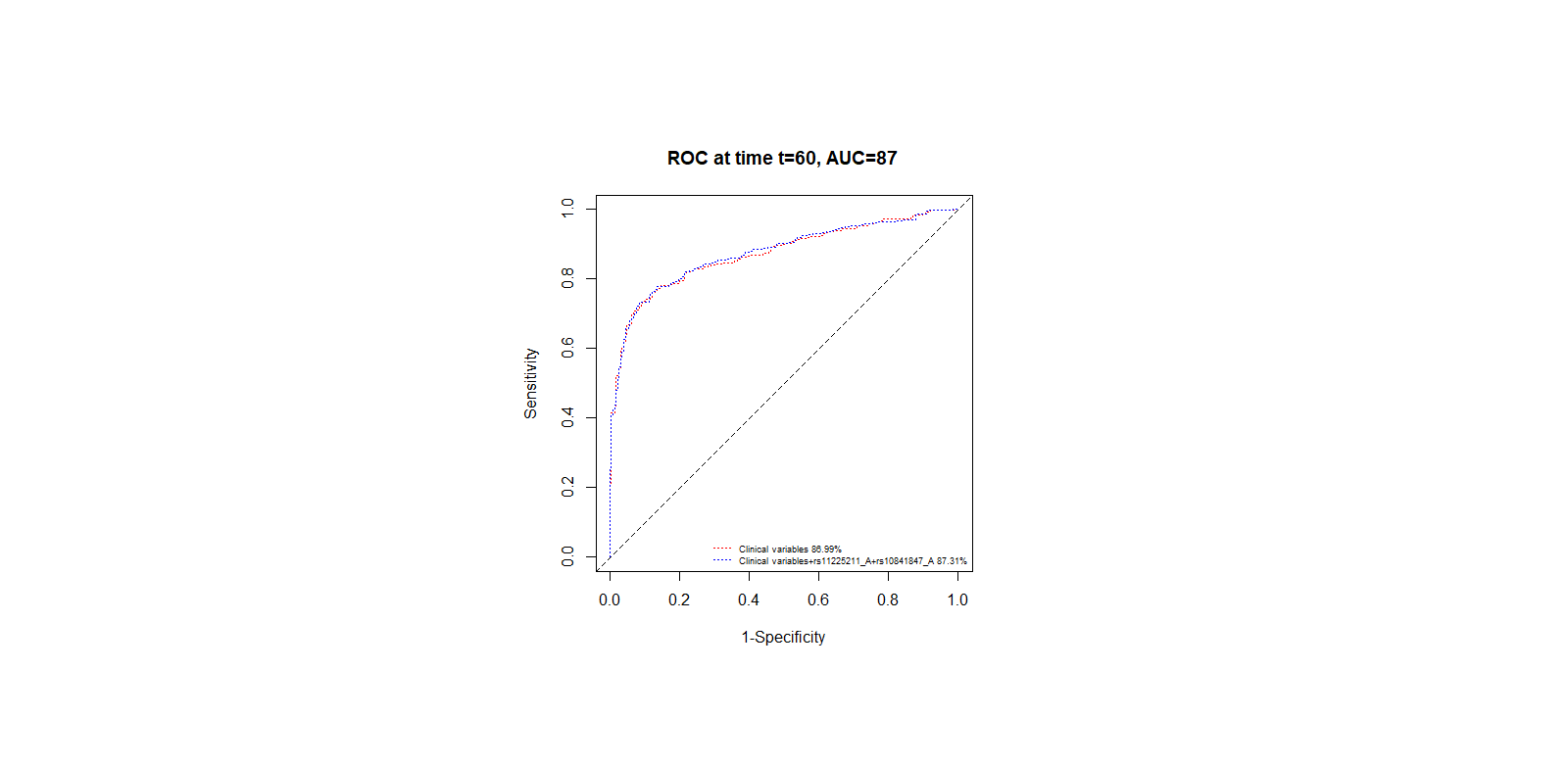


***P*=0.074**

**time t**

**Specificity**

**Sensitivity**

**a**

**b**

**c**

**d**

***P*=0.070**

ROC at time t=60, AUC=86.7

(b)

(a)

ROC at time t=60, AUC=86.9

**Supplementary Figure S4. ROC curve and time-dependent AUC estimation for five-year NSCLC survival predicted.** The ten-year NSCLS DSS prediction by time-dependent AUC estimation based on clinical variables plus protective alleles in the PLCO trial (**a**). The ten-year NSCLS DSS prediction by ROC curve based on clinical variables plus protective alleles in the PLCO trial (**b**). The ten-year NSCLS OS prediction by time-dependent AUC estimation based on clinical variables plus protective alleles in the PLCO trial (**c**). The ten-year NSCLS OS prediction by ROC curve based on clinical variables plus protective alleles in the PLCO trial (**d**). Abbreviations: ROC, receiver operating characteristic; AUC, area under receiver curve; NSCLC, non-small cell lung cancer; PLCO, Prostate, Lung, Colorectal and Ovarian Cancer Screening Trial; DSS, disease-specific survival; OS, overall survival.

**Supplementary Figure S5.** **Functional prediction of two independent SNPs in the DMAP-related pathway genes in the ENCODE data.** Location and functional prediction of *CLEC4E* rs10841847 (a). Location and functional prediction of *BIRC3* rs11225211 (b). The H3K27Ac, H3K4Me1, and H3K4Me3 tracks showed the genome-wide levels of enrichment of acetylation of lysine 27, the mono-methylation of lysine 4, and tri-methylation of lysine 4 of the H3 histone protein. DNase clusters track showed DNase hypersensitivity areas. Tnx factor track showed regions of transcription factor binding of DNA.


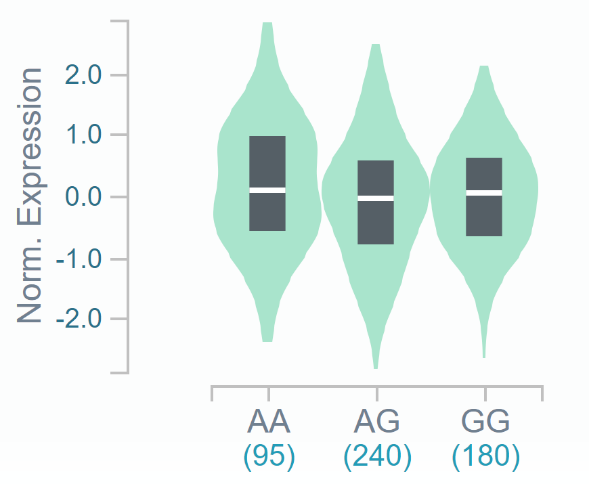

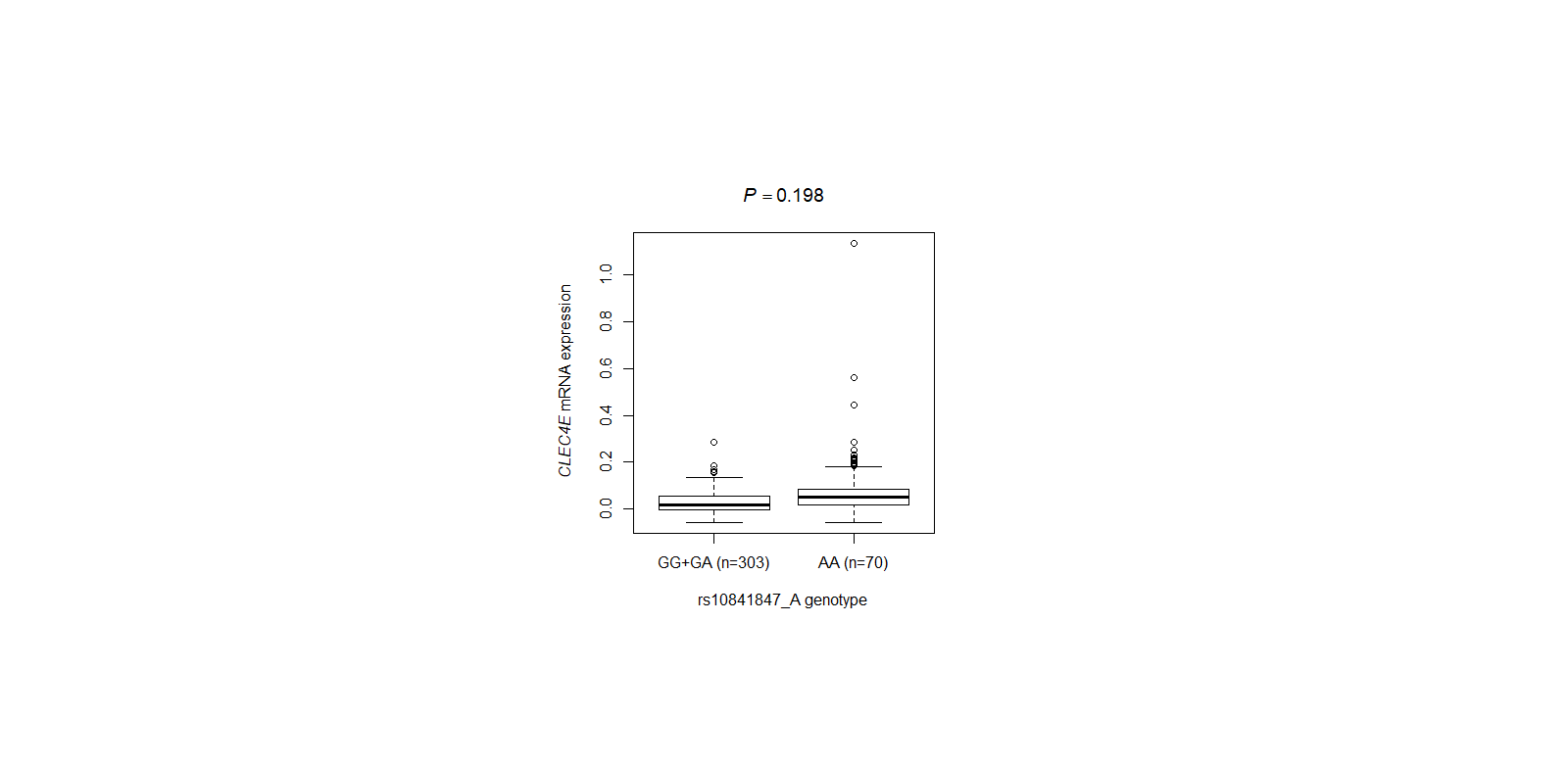


***P*=0.198**

***CLEC4E*mRNA expression**

**GG+GA**

**^(303)^**

**AA**

**^(70)^**


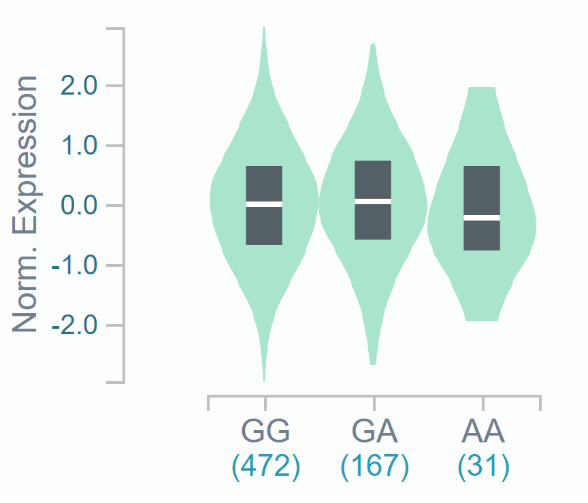


***CLEC4E* mRNA expressionAUC(t)**

**GG**

**^(472)^**

**AA**

**^(31)^**

**GA**

**^(167)^**

***P*=0.30**

**Lung**


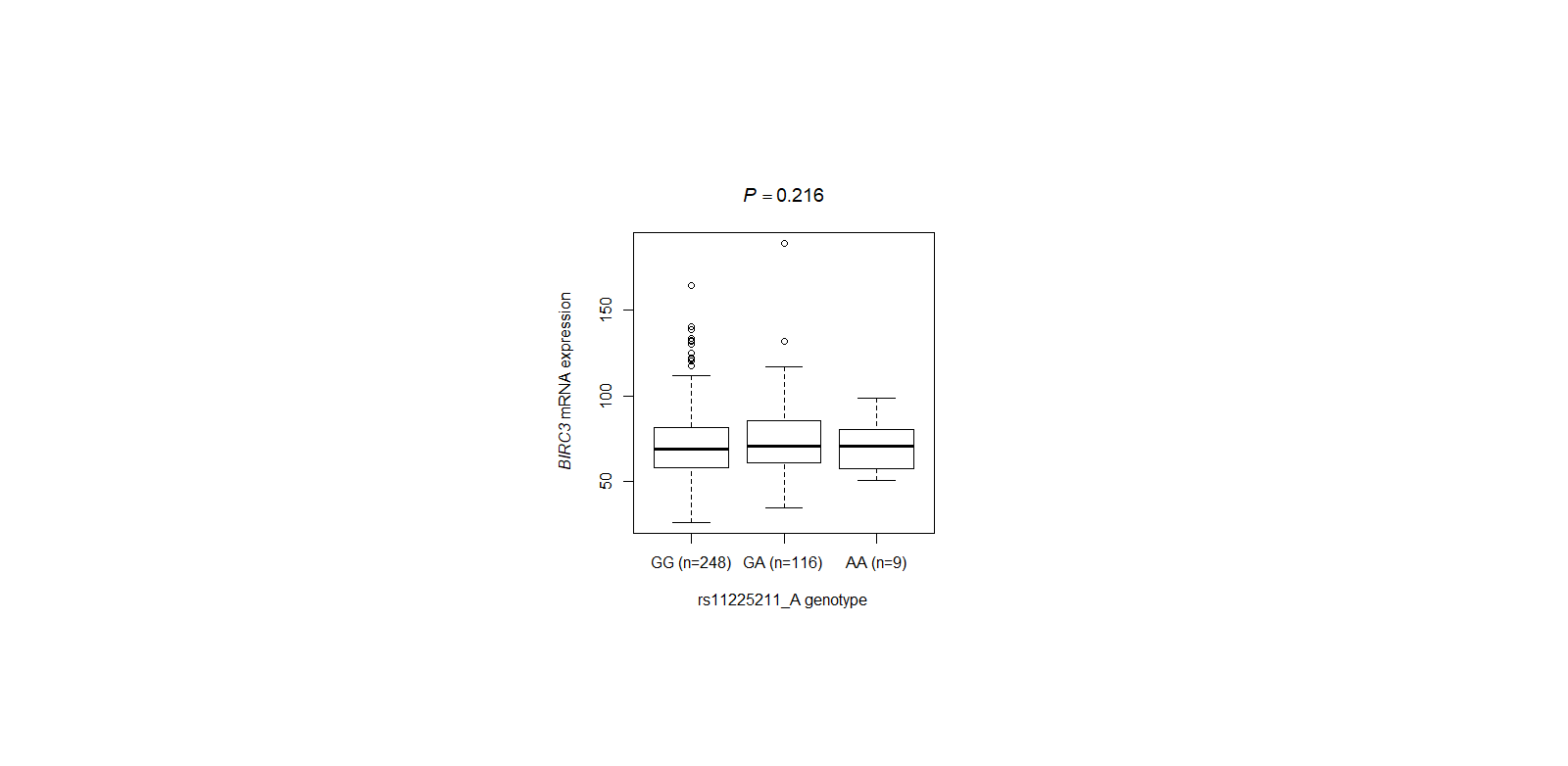


***P*=0.216**

**GG**

**^(248)^**

**GA**

**^(116)^**

**AA**

**^(9)^**

***BIRC3*mRNA expression**


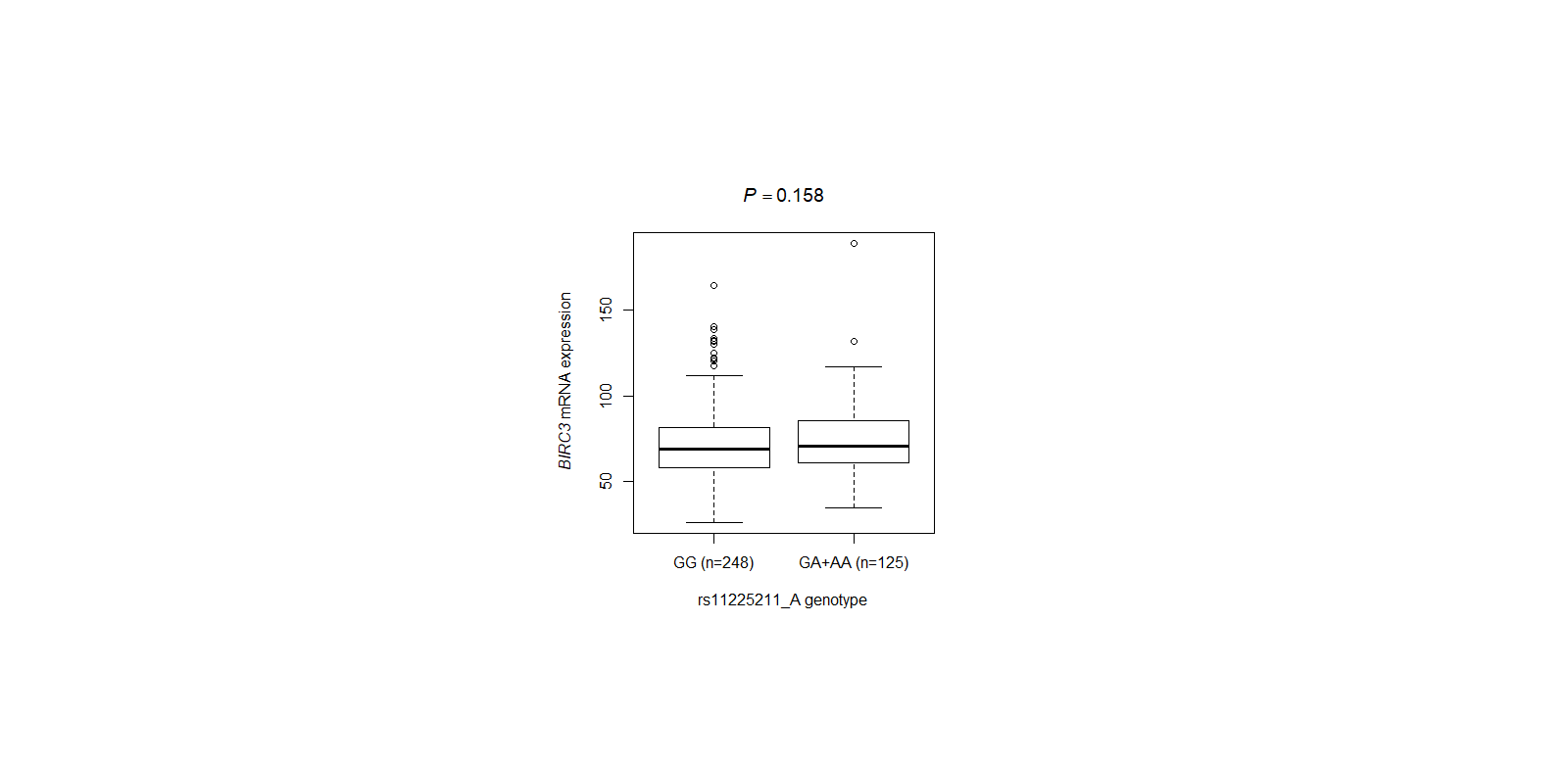


***P*=0.158**

**GG**

**^(248)^**

**GA+AA**

**^(125)^**

***BIRC3*mRNA expression**


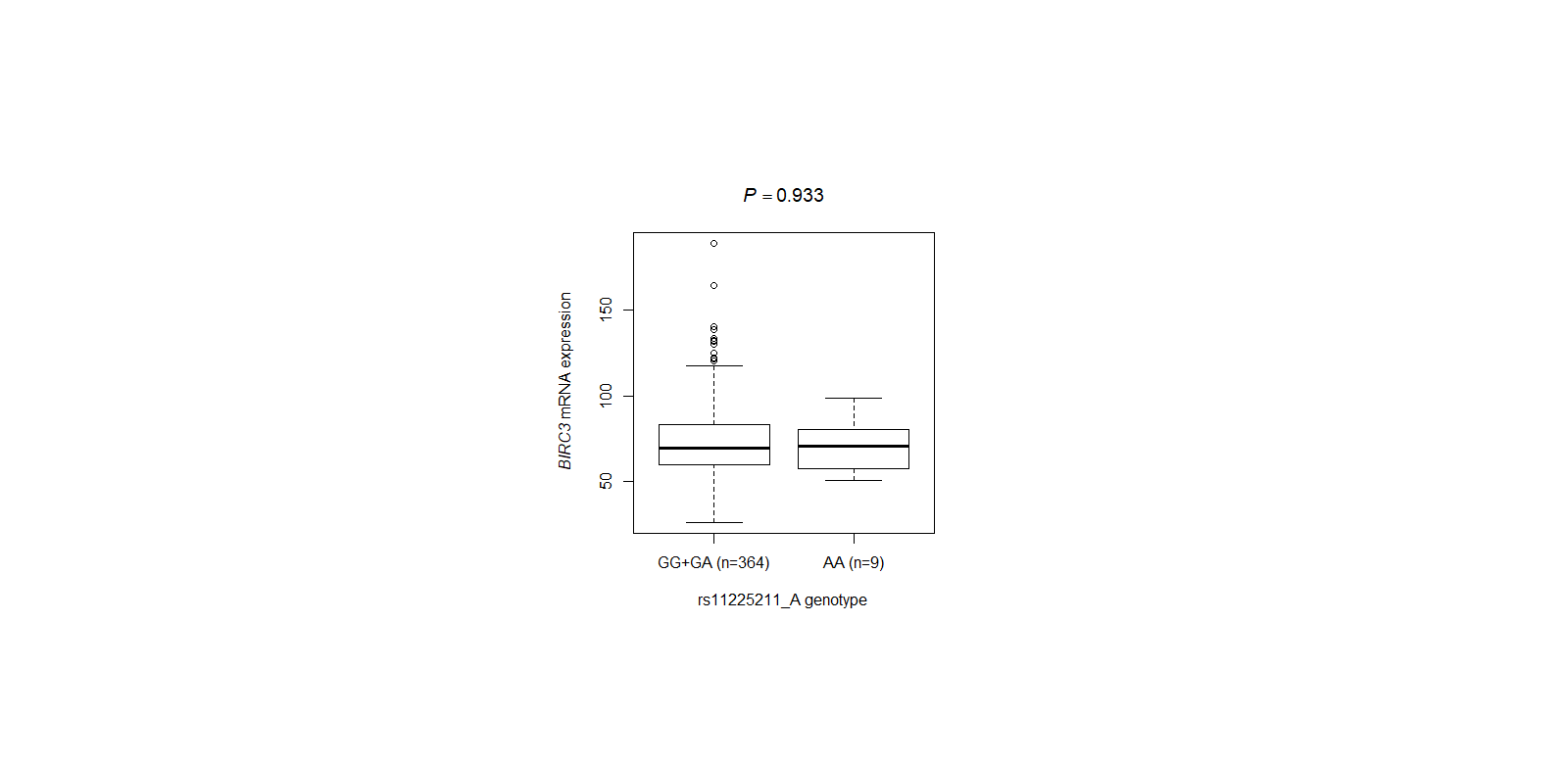


**GG+GA**

**^(364)^**

**AA**

**^(9)^**

***P*=0.933**

***BIRC3*mRNA expression**

***BIRC3*mRNA expression**

**Whole Blood**

**AA**

**^(95)^**

**AG**

**^(167)^**

**GG**

**^(472)^**

***P*=0.99**

**a**

**b**

**f**

**e**

**d**

**c**

***CLE4E* genotype**

***BIRC3* genotype**

***BIRC3* genotype**

***BIRC3* genotype**

***CLE4E* genotype**

***BIRC3* genotype**

**Supplementary Figure S6.** **The eQTLs analysis for *CLEC4E* rs10841847 and *BIRC3* rs11225211.** The correlation of rs10841847 genotypes and *CLEC4E* mRNA expression in the recessive model (a). The correlation of rs11225211 genotypes and *BIRC3* mRNA expression in the additive model (b), the dominant model (c), and the recessive model (d) from the 1000 Genomes Project. The correlation of rs10841847 genotypes and *CLEC4E* mRNA expression in normal lung tissues from the GTEx database (e). The correlation of rs11225211 genotypes and *BIRC3* mRNA expression in whole blood samples from the GTEx database (f). Abbreviations: eQTLs, expression quantitative trait loci; GTEx, Genotype-Tissue Expression project.


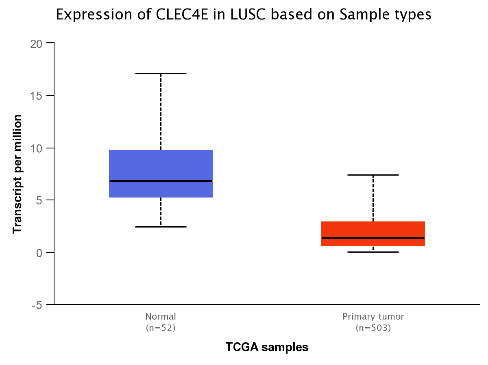

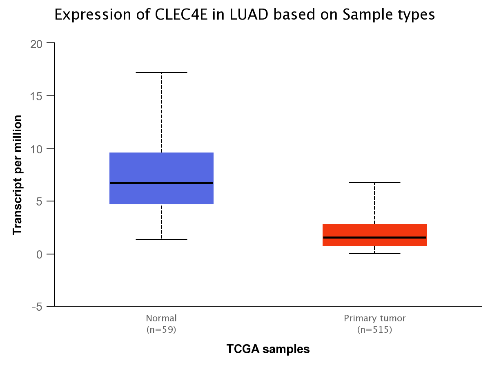


**a**

***P*=1.63x10^-12^**

**^***^**

**Normal**

**^(n=59)^**

**Primary tumor ^(n=515)^**

**Normal**

**^(n=52)^**

**Primary tumor ^(n=503)^**

**b**


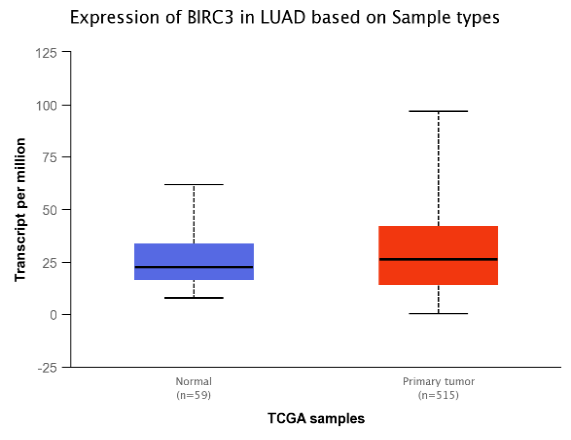


**Normal**

**^(n=59)^**

**Primary tumor ^(n=515)^**

***P*=4.54x10^-10^**

**^***^**

***P*=1.37x10^-4^**

**^***^**

**c**


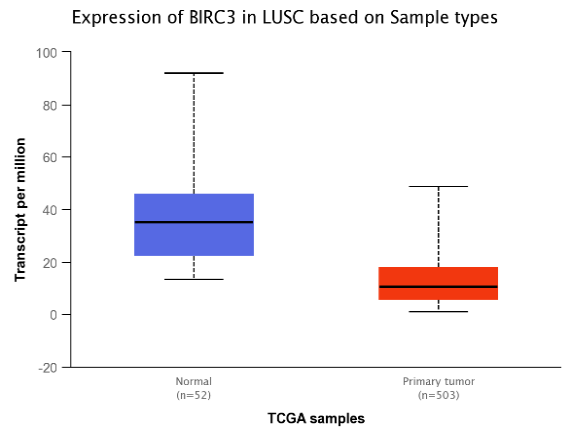


**d**

**Normal**

**^(n=52)^**

**Primary tumor ^(n=503)^**

***P*=1.20x10^-4^**

**^***^**


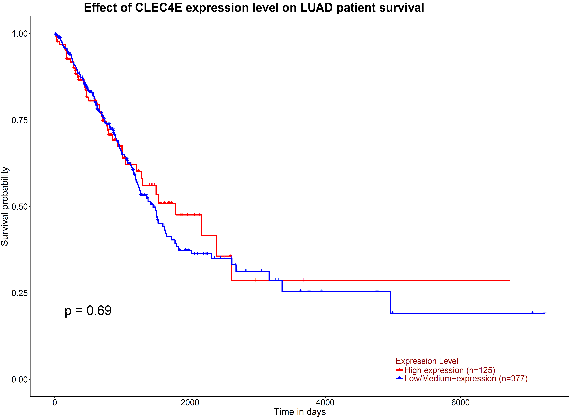

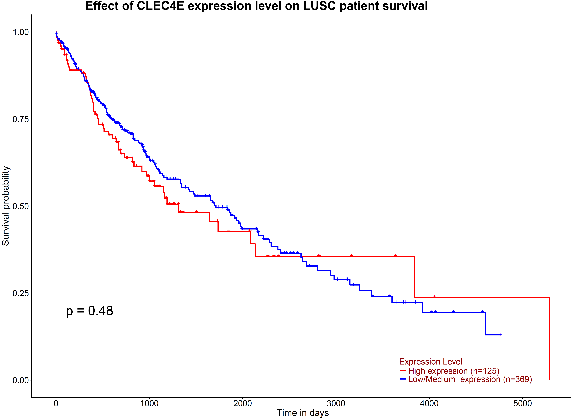

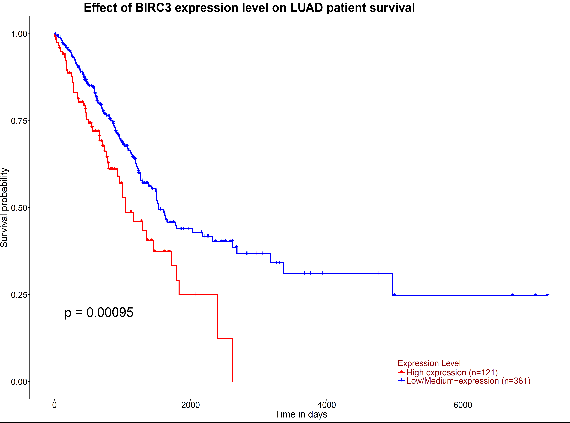

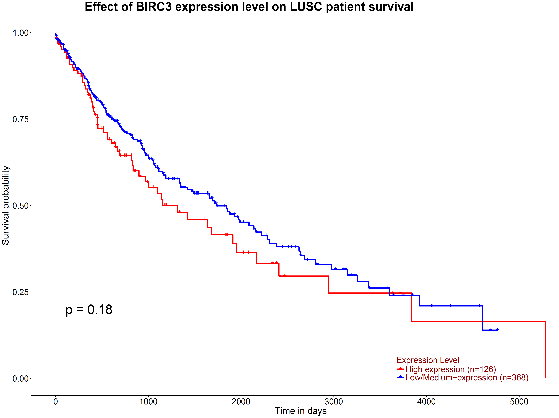


**e**

**f**

**g**

**h**


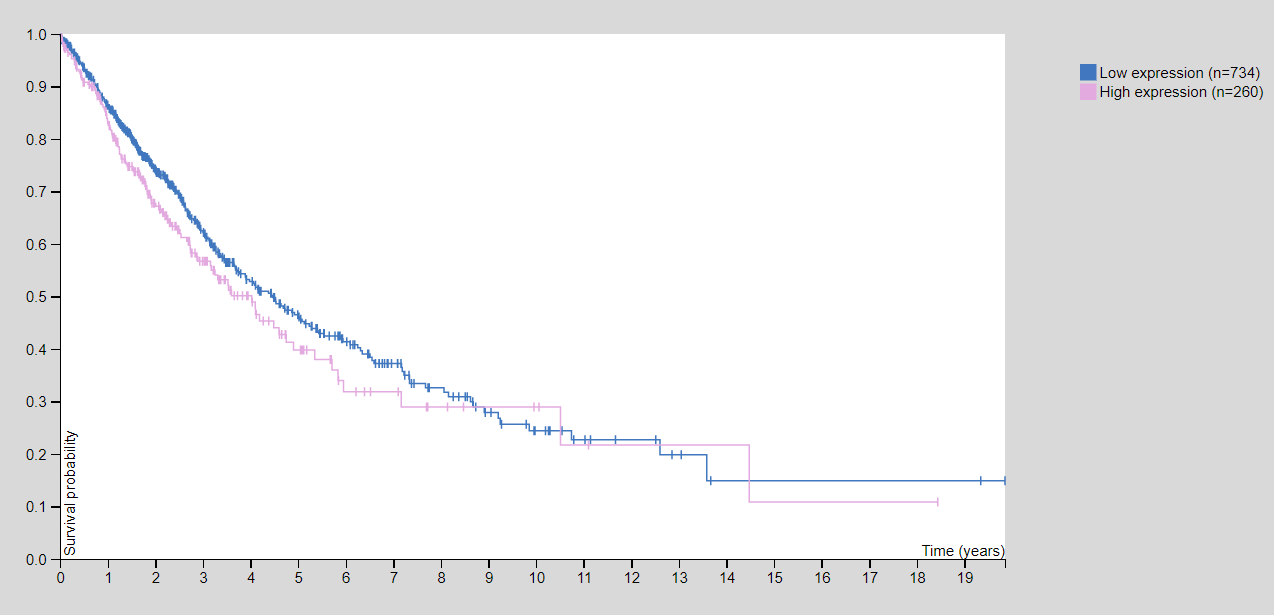

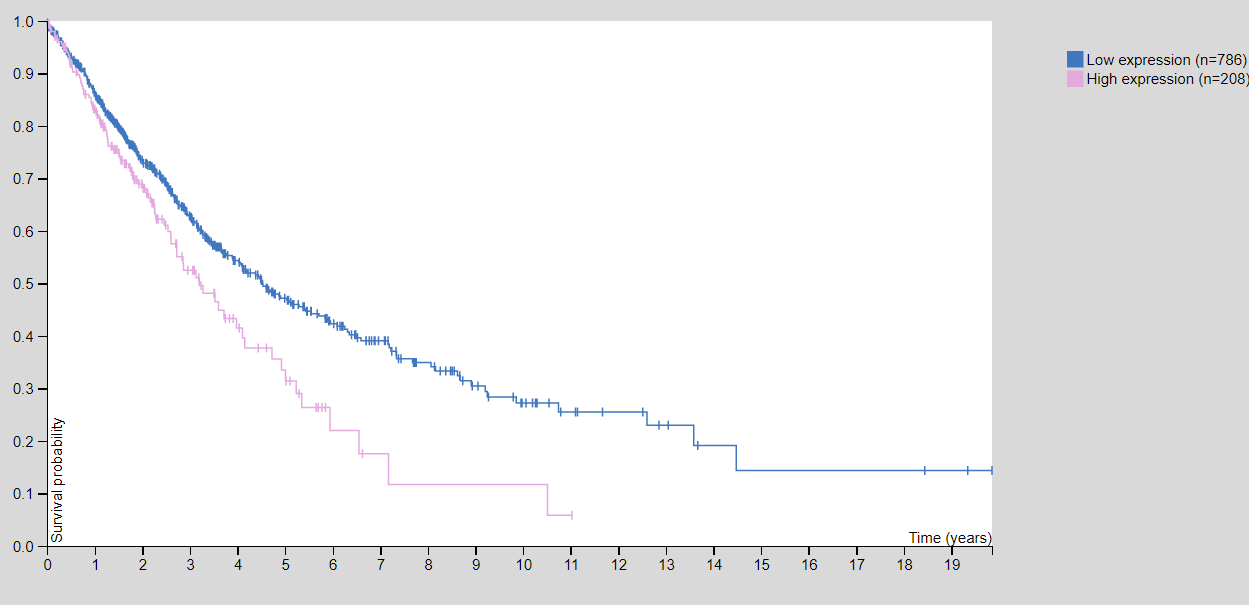


**Log-rank *P*=0.12**

**Log-rank *P*=0.004**

**i**

**j**

**Supplementary Figure S7. Differential mRNA expression analysis and survival analysis of *CLEC4E* and *BIRC3.*** The difference of *CLEC4E* mRNA expression between normal tissues and LUAD tissues in the TCGA database (**a**); The difference of *CLEC4E* mRNA expression between normal tissues and LUSC tissues in the TCGA database (**b**); The difference of *BIRC3* mRNA expression between normal tissues and LUAD tissues in the TCGA database (**c**); The difference of *BIRC3* mRNA expression between normal tissues and LUSC tissues in the TCGA database (**d**); *CLEC4E* mRNA expression showed no correlation with LUAD (**e**) and LUSC (**f**) survival probability in TCGA database. *BIRC3* mRNA expression showed significant correlation with LUAD (**g**) survival probability, but no correlation with LUSC (**h**) survival probability in TCGA database. *CLEC4E* mRNA expression showed no correlation with NSCLC survival probability in Human Protein Atlas database (**i**). *BIRC3* mRNA expression showed significant correlation with NSCLC survival probability in Human Protein Atlas database (**j**). Abbreviations: LUAD, Lung adenocarcinoma; TCGA, The Cancer Genome Atlas; LUSC, Lung squamous cell carcinoma; NSCLC, non-small cell lung cancer. The difference of mRNA expression and online Kaplan-Meier Survival Curve in the TCGA database was obtained from http://ualcan.path.uab.edu/cgi-bin/ualcan-res.pl. The online Kaplan-Meier Survival Curve in Human Protein Atlas database was obtained from https://www.proteinatlas.org/.
